# Deficiency of the splicing factor RBM10 limits EGFR inhibitor response in *EGFR*-mutant lung cancer

**DOI:** 10.1172/JCI145099

**Published:** 2022-07-01

**Authors:** Shigeki Nanjo, Wei Wu, Niki Karachaliou, Collin M. Blakely, Junji Suzuki, Yu-Ting Chou, Siraj M. Ali, D. Lucas Kerr, Victor R. Olivas, Jonathan Shue, Julia Rotow, Manasi K. Mayekar, Franziska Haderk, Nilanjana Chatterjee, Anatoly Urisman, Jia Chi Yeo, Anders J. Skanderup, Aaron C. Tan, Wai Leong Tam, Oscar Arrieta, Kazuyoshi Hosomichi, Akihiro Nishiyama, Seiji Yano, Yuriy Kirichok, Daniel S.W. Tan, Rafael Rosell, Ross A Okimoto, Trever G. Bivona

**Affiliations:** 1 Department of Medicine and; 2 Helen Diller Family Comprehensive Cancer Center, UCSF, San Francisco, California, USA.; 3 Division of Medical Oncology, Kanazawa University Cancer Research Institute, Kanazawa, Japan.; 4 Germans Trias i Pujol Research Institute and Hospital (IGTP), Badalona, Spain.; 5 Department of Physiology, UCSF, San Francisco, California, USA.; 6 Foundation Medicine Inc., Cambridge, Massachusetts, USA.; 7 Department of Pathology, UCSF, San Francisco, California, USA.; 8 Genome Institute of Singapore, Agency for Science, Technology and Research, Singapore.; 9 Division of Medical Oncology, National Cancer Centre Singapore, Singapore.; 10 Cancer Science Institute of Singapore, National University of Singapore, Singapore.; 11 Thoracic Oncology Unit, National Cancer Institute (INCan), México City, Mexico.; 12 Department of Bioinformatics and Genomic, Kanazawa University, Kanazawa, Japan.; 13 Chan-Zuckerberg Biohub, San Francisco, California, USA.

**Keywords:** Oncology, Therapeutics, Cancer gene therapy, Lung cancer

## Abstract

Molecularly targeted cancer therapy has improved outcomes for patients with cancer with targetable oncoproteins, such as mutant EGFR in lung cancer. Yet, the long-term survival of these patients remains limited, because treatment responses are typically incomplete. One potential explanation for the lack of complete and durable responses is that oncogene-driven cancers with activating mutations of *EGFR* often harbor additional co-occurring genetic alterations. This hypothesis remains untested for most genetic alterations that co-occur with mutant *EGFR*. Here, we report the functional impact of inactivating genetic alterations of the mRNA splicing factor RNA-binding motif 10 (*RBM10*) that co-occur with mutant *EGFR*. RBM10 deficiency decreased EGFR inhibitor efficacy in patient-derived *EGFR*-mutant tumor models. RBM10 modulated mRNA alternative splicing of the mitochondrial apoptotic regulator Bcl-x to regulate tumor cell apoptosis during treatment. Genetic inactivation of *RBM10* diminished EGFR inhibitor–mediated apoptosis by decreasing the ratio of (proapoptotic) Bcl-xS to (antiapoptotic) Bcl-xL isoforms of Bcl-x. RBM10 deficiency was a biomarker of poor response to EGFR inhibitor treatment in clinical samples. Coinhibition of Bcl-xL and mutant EGFR overcame the resistance induced by RBM10 deficiency. This study sheds light on the role of co-occurring genetic alterations and on the effect of splicing factor deficiency on the modulation of sensitivity to targeted kinase inhibitor cancer therapy.

## Introduction

Lung cancer is the leading cause of cancer mortality among men and women, comprising almost 25% of all cancer deaths (1). There has been significant progress in the treatment of lung cancer and many other cancer types in the past 10 years with the advent of precision medicine that leverages tumor molecular and genetic profiling coupled with molecularly targeted cancer therapy. As 1 paradigm-defining example of precision medicine, activating mutations in the *EGFR* are associated with high response rates to EGFR-directed tyrosine kinase inhibitors (TKIs) in non–small cell lung cancer (NSCLC) (1). The most common TKI-sensitizing mutations in the *EGFR* are deletions in exon 19 that affect the LREA motif and substitutions in exon 21 (*L858R*), which together account for more than 90% of all *EGFR* mutations in lung adenocarcinoma (LA) (2). Although these canonical *EGFR* mutations typically confer sensitivity to EGFR TKIs, approximately 20%–30% of patients exhibit either primary refractory disease (intrinsic resistance) or a limited response (such as less than 30% tumor regression by Response Evaluation Criteria in Solid Tumors [RECIST], version 1.1 criteria; ref. 3) followed by disease progression (4–7). While several mechanisms of intrinsic resistance have been reported (8–13), the mechanisms underlying the distinct clinical scenario of limited tumor response followed by earlier tumor progression during initial EGFR TKI treatment are less well defined. This is perhaps highlighted through clinical trial observations that patients harboring the *EGFR L858R* mutation experienced shorter progression-free survival (PFS) with identical EGFR inhibitor treatment compared with patients with *EGFR* exon 19 deletions for reasons that are not well understood (4–7).

The use of next-generation sequencing (NGS) platforms that profile large panels of cancer-relevant genes has shown that *EGFR*-mutant tumors often harbor additional co-occurring genetic alterations, both before and after therapy (14–16). One hypothesis arising from this observation is that certain co-occurring alterations present in an *EGFR*-mutant tumor could modulate sensitivity to EGFR TKI treatment and explain, in part, the variable magnitude and duration of antitumor treatment responses in patients. With a few important exceptions such as genetic alterations of *TP53* (14, 15), the functional impact (if any) of most of these co-occurring genetic alterations on tumor growth or treatment sensitivity is not well established.

One mechanism by which co-occurring genetic alterations could affect therapeutic sensitivity to EGFR TKI treatment is via the modulation of tumor cell apoptosis (17). The Bcl-2 family of proteins regulates the intrinsic pathway of mitochondria-mediated apoptosis (18) and comprises both proapoptotic and antiapoptotic components. The proapoptotic arm of this pathway is often activated by cancer treatment, which initiates the depolarization of the mitochondrial outer membrane potential and release of cytochrome c into the cytoplasm to form the apoptosome, which subsequently activates caspase-3 and caspase-7, which are effector caspases (19). In *EGFR*-mutant tumors, upregulation of the proapoptotic Bcl-2 family protein BIM is an important event that is required for EGFR inhibitor–induced apoptosis (17). Genetic loss of *BIM* can limit the EGFR inhibitor therapeutic response (12, 13). Beyond BIM modulation, how *EGFR*-mutant tumor cells regulate the apoptotic response during EGFR-targeted therapy remains incompletely defined.

mRNA alternative splicing is 1 mechanism used by cells to generate the phenotypic and functional diversity that affects a range of cellular behaviors (20, 21). Recent studies identified a role for mRNA-splicing factors in oncogenesis in hematologic malignancies (22, 23). Recent reports also indicate the presence of genetic alterations in mRNA splicing genes in solid malignancies (24). For instance, The Cancer Genome Atlas (TCGA) LA profiling analysis showed mutation of the splicing factor RNA-binding motif 10 (*RBM10*) at a relatively high frequency (25, 26). The potential role of these RBM10 genetic alterations in LA is not well defined. RBM10 can regulate mRNA alternative splicing and may act as a tumor suppressor in some contexts (27–29). Here, we investigated the open question of whether and how mutations in splicing factors such as *RBM10* contribute to lung cancer pathogenesis or modulate sensitivity to oncoprotein-targeted kinase inhibitor therapy.

## Results

### Identification of potential genetic modifiers of EGFR inhibitor response highlights RBM10.

**Figure 1:**
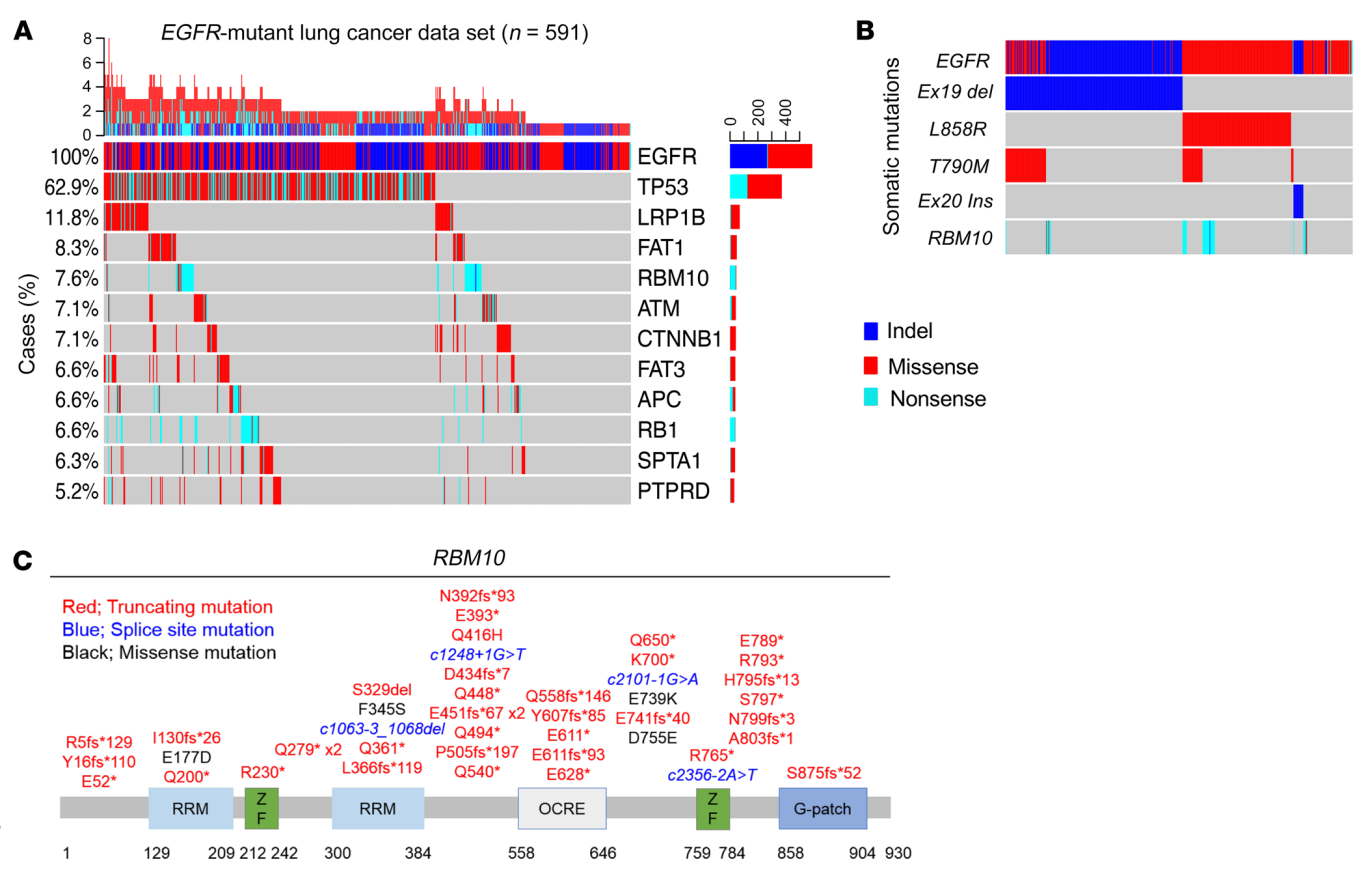
*RBM10* mutations co-occur with *EGFR* mutations in LA. (**A**) Targeted NGS of 591 *EGFR*-mutant LA tumors using a panel of 324 cancer-related genes (median coverage depth = 500×). Co-occurring alterations that occurred in at least 5% of *EGFR* mutation–positive cases are shown. (**B**) *RBM10* alterations were compared across each *EGFR*-mutant subtype. (**C**) Mutations in the *RBM10* protein-coding sequence (splice site mutations: blue; truncating mutations: red; missense mutations: black). RRM, RNA recognition motifs; ZF, zinc finger; G-patch, glycine patch.

Oncogenic *EGFR*-activating mutations are associated with high initial response rates to EGFR-directed therapy in human LA (1). However, 20%–30% of patients with *EGFR* mutations will either not respond with demonstrable (i.e., >30%) tumor regression or develop relatively early tumor progression following an initial incomplete response (3) to EGFR TKI treatment (4–7). Tumor genetic heterogeneity that is present prior to therapy could contribute to this incomplete initial response or early emergence of resistance. In order to identify genetic modifiers of therapeutic responses in *EGFR*-mutant LA, we performed targeted next-generation sequencing (NGS) of 324 cancer-relevant genes in 591 *EGFR*-mutant LA human tumors (median coverage depth = 500x) (see Methods). Through this analysis, we noted frequent truncating mutations in the *RBM10* gene (7.6%) (Figure 1A). A similar frequency of *RBM10* truncating mutations was present in the MSK-IMPACT (Memorial Sloan Kettering – Integrated Mutation Profiling of Actionable Cancer Targets) *EGFR*-mutant LA data set (8.0%) (Supplemental Figure 1A; supplemental material available online with this article; https://doi.org/10.1172/JCI145099DS1) (30). Interestingly, we observed that 92% of *RBM10* alterations present in our *EGFR-*mutant tumor data set did not co-occur with a known EGFR TKI resistance–associated mutation such as *EGFR T790M* or *C797S* or *MET* gene amplification (Supplemental Figure 1B). This suggests a potential modifying role for *RBM10* mutations in these *EGFR*-mutant tumors outside of known genetic mechanisms of resistance (Figure 1B). Consistent with prior observations, *RBM10* mutations in our data set primarily resulted in premature truncation of the protein-coding sequence, suggesting a loss-of-function phenotype (Figure 1C) (24–25). In general, *RBM10* mutations were more often subclonal compared with the founder *EGFR* mutation (Fisher’s exact test *P =* 0.02, Supplemental Figure 1C) in *EGFR*-mutant tumors (31). Since there is no known functional impact of *RBM10* on *EGFR*-mutant lung cancer treatment, we tested the hypothesis that *RBM10* inactivation may modulate EGFR TKI sensitivity in *EGFR*-mutant LA.

**Figure 2:**
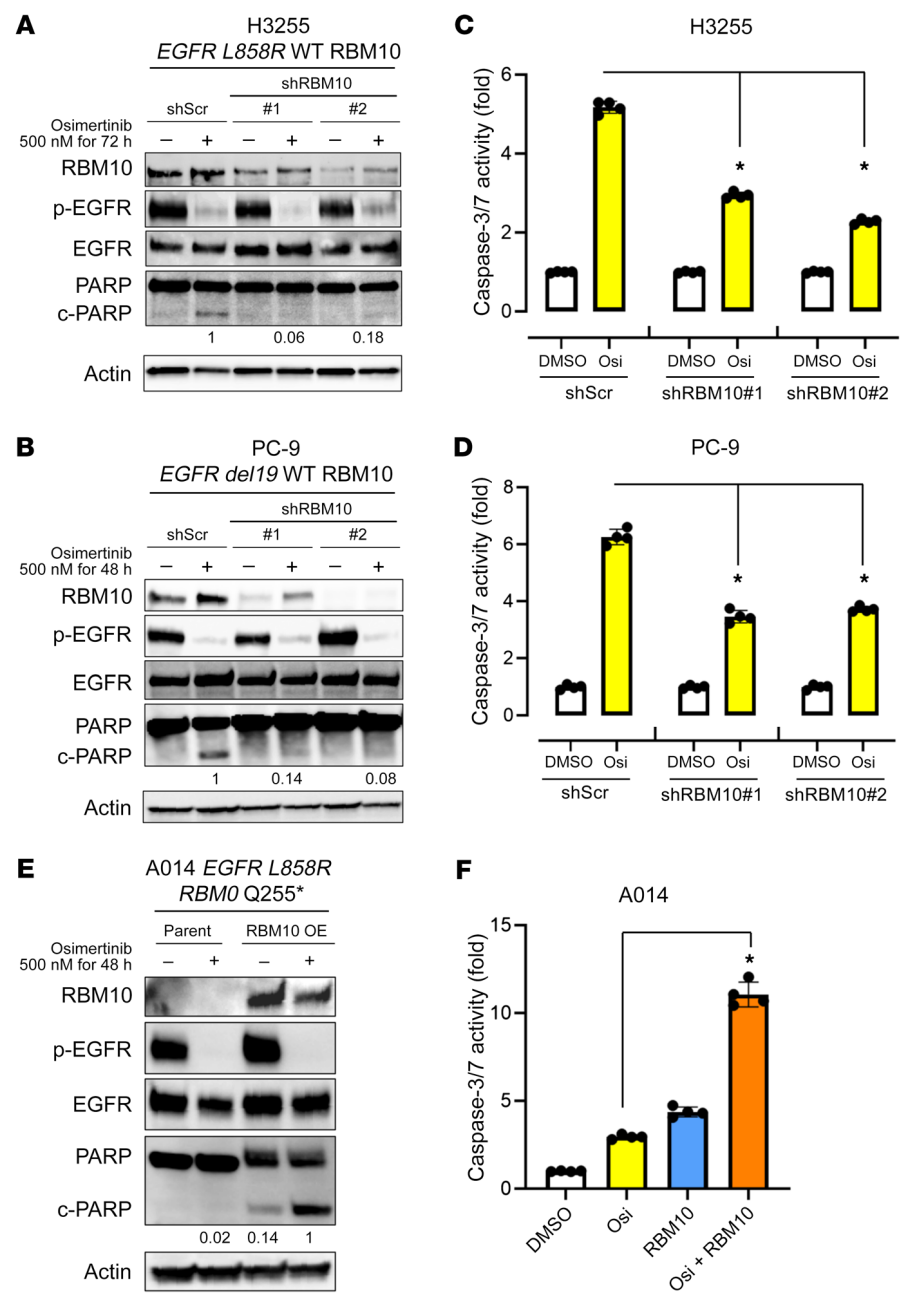
RBM10 modulates the apoptotic response to osimertinib in *EGFR*-mutant LA. (**A**–**D**) H3255 and PC-9 (mutant *EGFR* and WT *RBM10*) cells expressing sh*RBM10* or the shScr control were treated with the third-generation EGFR inhibitor osimertinib (500 nM) or DMSO for 48–72 hours. Western blot analysis of the indicated proteins from cellular protein extracts was normalized to actin. (**A** and **B**) Quantification of cleaved PARP was determined by signal densitometry. (**C** and **D**) The apoptotic response was assessed using a Caspase-Glo 3/7 assay. Each bar represents the mean ± SEM of the FC after normalization to the DMSO control. (**E** and **F**) RBM10-deficient A014 (*EGFR*-mutant and RBM10 Q255*) cells with genetic reconstitution of WT *RBM10* were treated with osimertinib (500 nM) for 48 hours. Western blotting of the indicated lysates was normalized to actin (**E**). Caspase 3/-7 activity was measured using a Caspase-Glo 3/7 assay. Each bar represents the mean ± SEM of the FC after normalization to DMSO control. (**F**). Data represent 3 independent experiments. *P < 0.05, by 1-way ANOVA. Osi, osimetertinib.

To test this hypothesis, first we performed functional genetic experiments by *RBM10* knockdown (KD) and overexpression (OE) in patient-derived lung cancer cell lines to assess cell viability and apoptotic responses to the EGFR kinase inhibitor osimertinib, which we used at clinically relevant concentrations (32). We observed increased cell viability during osimertinib treatment upon genetic silencing of *RBM10* with 2 independent shRNAs in the patient-derived LA cell lines H3255 (*EGFR L858R*) and PC-9 (*EGFR del19*) compared with the control (Supplemental Figure 2, A–F). To assess whether RBM10 expression could modulate apoptosis in *EGFR*-mutant lung cancer, we first assessed cleavage of the apoptotic biomarker poly (ADP-ribose) polymerase (PARP) in H3255 (*EGFR L858R*/*RBM10* WT) and PC-9 (*EGFR del19*/*RBM10* WT) cells. Indeed, after osimertinib treatment, we found that cleaved PARP levels were decreased in *RBM10*-KD cells compared with levels in control cells (Figure 2, A and B). Additionally, the activities of the key apoptotic effectors caspases 3 and -7 were similarly decreased upon *RBM10* KD in H3255 and PC-9 cells treated with osimertinib (Figure 2, C and D). We next studied a recently established *EGFR L858R*–mutant patient-derived cell line (A014) that harbors a co-occurring *RBM10* truncating mutation and has relative resistance to EGFR inhibitors, showing minimal apoptosis upon EGFR TKI treatment (Figure 2, E and F) (16). We reconstituted WT *RBM10* into these RBM10-deficient A014 cells and observed enhanced apoptosis upon osimertinib treatment, as measured by cleaved PARP levels and caspase 3 and -7 activity (Figure 2, E and F). These data highlight the RBM10 functional deficiency that characterizes the *RBM10*-mutant A014 patient-derived model. In the absence of osimertinib treatment, *RBM10* KD did not initiate tumor formation in noncancerous Beas2B human bronchial epithelial cells lacking canonical driver mutations (Supplemental Figure 3A), nor did *RBM10* KD significantly enhance tumor growth or proliferation in H3255 or PC-9 patient-derived *EGFR*-mutant LAs in in vivo subcutaneous tumor models (Supplemental Figure 3, C–F). The baseline apoptosis levels, as measured by cleaved caspase 3 and TUNEL IHC, did not significantly differ between shScramble (shScr) and sh*RBM10* conditions in H3255 or PC-9 in vivo subcutaneous tumors (Supplemental Figure 3, E and F). We confirmed that tumor growth was not impacted by RBM10 loss using H3255 and PC-9 *RBM10* CRISPR-KO cells implanted and grown in vivo for over 2 months (Supplemental Figure 4, A–D). According to TCGA RPPA data, cleaved caspase 7 levels do not differ between *RBM10*-mutant and WT tumors without EGFR TKI treatment (Supplemental Figure 5). Collectively, these findings suggest that RBM10 expression affects the therapeutic sensitivity to EGFR-targeted therapy by modulating the apoptotic response, without a substantial impact on tumor cell proliferation or tumor initiation or growth in the absence of either oncogenic *EGFR* or EGFR inhibitor treatment in these various systems.

**Figure 3:**
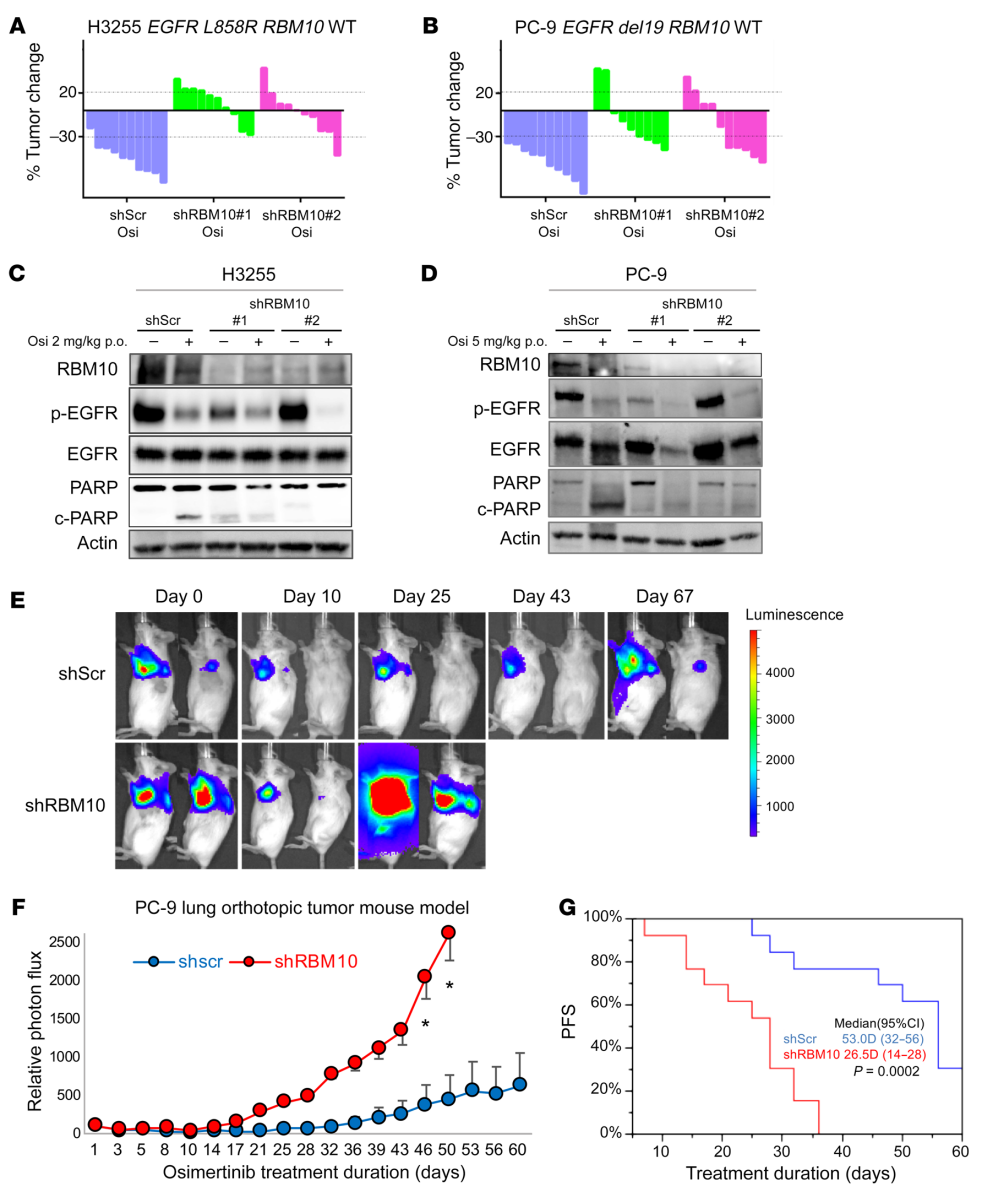
RBM10 deficiency limits the therapeutic efficacy of EGFR TKIs. (**A** and **B**) Waterfall plots representing immunodeficient mice bearing H3255 (**A**) or PC-9 (**B**) tumor xenografts expressing either shScr control or sh*RBM10*. Mice were treated with 2 mg/kg (H3255) or 5 mg/kg (PC-9) osimertinib once daily over 14 days (*n =* 10 tumors per treatment cohort). Percentage changes in tumor volume compared with baseline volume (day 0) for individual tumor xenografts are shown. (**C** and **D**) H3255 and PC-9 tumor xenograft explants demonstrating the effect of *RBM10* KD on PARP cleavage in mice treated with osimertinib or vehicle for 14 days. One tumor of representative size from each group was harvested 4 hours after the indicated treatments on day 15, and subsequent analyses of the indicated proteins was performed by Western blotting. (**E**–**G**) PC-9 cells expressing either shScr or sh*RBM10* in a validated orthotopic lung tumor model were treated with 5 mg/kg osimertinib once daily for 60 days. Representative bioluminescence images (**E**) and mean relative photon flux (**F**) are shown. *P < 0.05. (**G**) PFS comparing the PC-9 shScr control and PC-9 sh*RBM10* mice (*P* = 0.0002, Wilcoxon test).

We next tested whether co-occurring *RBM10* inactivation in *EGFR*-mutant tumors contributes to EGFR inhibitor resistance in vivo. We treated mice bearing H3255 or PC-9 tumor xenografts with osimertinib or vehicle and found that mice with tumors in which *RBM10* was silenced had decreased osimertinib sensitivity compared with mice bearing shScr control–expressing tumors (Figure 3, A and B, and Supplemental Figure 3, C and D). Using clinical response criteria (33), we observed that *EGFR*-mutant tumors harboring sh*RBM10* had a decreased depth and frequency of response (Tables 1 and 2). Consistent with our in vitro findings, we found decreased PARP cleavage in osimertinib-treated tumors explanted from mice bearing tumors in which *RBM10* was silenced compared with control tumors (Figure 3, C and D, and Supplemental Figure 6, A and B).

In order to model PFS in *EGFR*-mutant LA, we leveraged our experience with an in vivo luciferase-based orthotopic lung cancer model (34, 35). We surgically implanted luciferase-labeled PC-9 cells expressing either the shScr control or sh*RBM10* and treated these mice with osimertinib. We observed earlier initial tumor progression in mice bearing *RBM10*-KD tumors compared with controls in osimertinib-treated mice (Figure 3, E–G) (Wilcoxon test *P* = 0.0002, *n =* 13/group). Thus, RBM10 suppression limited the initial response to EGFR-targeted therapy in multiple patient-derived in vitro and in vivo systems.

### Clinical impact of RBM10 downregulation in advanced-stage EGFR-mutant lung cancer treated with an EGFR TKI.

**Figure 4:**
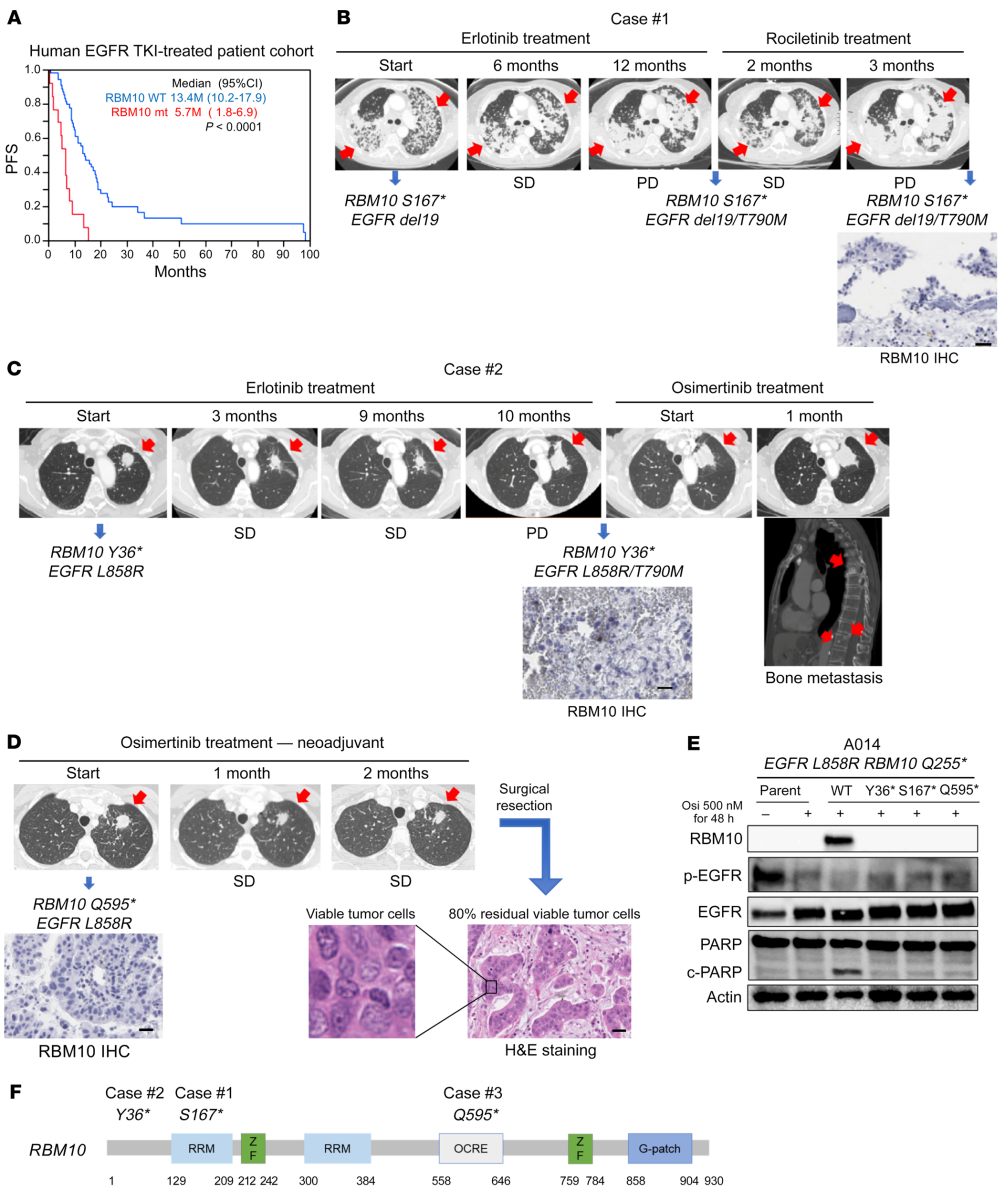
RBM10 deficiency is a biomarker of poor EGFR TKI responses in human *EGFR*–mutant lung cancer. (**A**) A human EGFR TKI–treated patient cohort (*n =* 70) was stratified into WT *RBM10* and *RBM10*-mutant (mt) cohorts. PFS (*P* value was determined by Wilcoxon test) in WT *RBM10* and *RBM10*-mutant cohorts is shown. (**B**–**D**) Somatic alterations were detected by NGS panel analysis of the tumor DNA from the patients. (**B**) Case 1 involved a patient harboring co-occurring mutations in *EGFR del19* and RBM10 S167* prior to EGFR inhibitor treatment. This patient had SD on 6 months of erlotinib therapy, followed by early progression on third-generation EGFR TKI rociletinib with the acquisition of an *EGFR* *T790M* mutation. (**C**) In case 2, the patient had co-occurring *EGFR L858R* and RBM10 Y36* mutations and had SD during 10 months of erlotinib treatment. Following progression on erlotinib, the patient had progressive local and metastatic disease on osimertinib. (**D**) Case 3 involved a patient enrolled in a neoadjuvant osimertinib clinical trial and found to harbor co-occurring *EGFR L858R* and RBM10 Q595* mutations. Following 2 months of osimertinib treatment, radiographic measurements indicated SD, and pathologic evaluation of the resected tumor specimen showed 80% viable tumor cells by H&E staining. RBM10 protein expression by IHC in patient-derived specimens obtained at either the time of progression (cases 1 and 2) or before neoadjuvant (case 3) EGFR TKI therapy are shown at ×200 magnification. Scale bars: 100 μm (**B**–**D**). (**E**) Immunoblot analysis of A014 (RBM10 Q255*) cells transfected with constructs overexpressing WT or mutant *RBM10* forms (Y36*, S167*, Q595*). Cells were treated with osimertinib (500 nM) or DMSO for 48 hours, and Western blot analysis was performed on cellular extracts. (**F**) Engineered RBM10 Y36*, RBM10 S167*, and RBM10 Q595* mutations are shown. Data represent 3 independent experiments.

Our preclinical data showed that low levels of RBM10 limited the response to EGFR TKI treatment. Therefore, we investigated the relationship between RBM10 expression levels and the EGFR TKI treatment response in human advanced-stage (IIIB/IV), *EGFR*-mutant NSCLC. We performed quantitative real-time PCR (qRT-PCR) analysis of *RBM10* in a panel (*n =* 88) of clinically annotated *EGFR*-mutant tumors obtained from patients treated with EGFR inhibitors. Although we were unable to perform direct genomic sequencing of *RBM10* in this clinical cohort, we and others have observed a significant (*P* < 0.01) association between *RBM10* mRNA expression and *RBM10* mutation status, wherein *RBM10* mutations are associated with decreased *RBM10* mRNA expression (Supplemental Figure 4C) (36). We stratified a total of 88 patients with *EGFR* mutations according to *RBM10* mRNA expression in quartiles and found that the patients whose tumors expressed lower levels of *RBM10* progressed earlier on EGFR TKI therapy (Wilcoxon test *P* = 0.0214) (Supplemental Figure 7A). Available clinical parameters were examined, and no significant differences in RBM10 were found between the groups stratified by these clinical parameters (Supplemental Table 1). We next analyzed PFS on the basis of *RBM10* mutation status. The *RBM10*-mutant cohort showed significantly shorter PFS compared with the WT *RBM10* group during EGFR TKI treatment (Wilcoxon test *P* < 0.0001) (Figure 4A). Decreased PFS in the *RBM10*-mutant group was associated with a decreased initial tumor response (*P =* 0.0041) (Table 3). We examined available co-mutation data and found no significant associations between these comutations and *RBM10* status (Supplemental Table 2). Our in vivo mouse model findings (Figure 3), coupled with these clinical observations, suggest that loss of RBM10 can limit the initial response to EGFR-targeted therapy by suppressing tumor cell apoptosis, leading to worse clinical outcomes for patients with *EGFR*-mutant LA.

### Clinicogenomic validation of mutant RBM10 in EGFR-mutant lung cancer.

We further assessed the clinical relevance of *RBM10* mutations in patients. We queried a database of a distinct UCSF-based cohort to identify patients with *EGFR*-mutant lung cancer whose tumors harbored co-occurring *RBM10* mutations. We reasoned that loss-of-function genetic alterations in *RBM10* that dampen the apoptotic response to EGFR inhibitor therapy could enhance cancer cell survival in patients with *EGFR-*mutant lung cancer. We confirmed that the truncating mutations in the UCSF cohort decreased RBM10 protein expression by IHC in patient-derived specimens obtained at either the time of progression on EGFR TKI treatment (cases 1 and 2) or following neoadjuvant EGFR TKI treatment (case 3; NCT03433469). The case 1 patient harbored the co-occurring mutations *EGFR del19* and RBM10 S167* prior to EGFR inhibitor treatment. This patient’s tumor showed only stable disease (SD) on erlotinib therapy (6 months), followed by early progression that coincided with the acquisition of the drug-resistant *T790M* mutation of *EGFR* (Figure 4B). In a second patient (case 2) with co-occurring *EGFR* *L858R* and RBM10 Y36* mutations, again we only observed SD during erlotinib treatment (10 months) (Figure 4C). Biopsy at the time of progression confirmed low RBM10 expression (Figure 4C). Osimertinib was initiated in this case, and there was immediate disease progression despite this treatment (Figure 4C). These clinical data are consistent with most of our experimental findings showing that genetic inactivation of *RBM10* limits the initial apoptotic response in *EGFR*-mutant tumor cells. In certain cases (e.g., case 1), RBM10 loss may mediate the escape from tumor cell apoptosis during initial treatment and before the subsequent emergence of acquired resistance mutations such as *EGFR T790M* that drive tumor cell proliferation.

To associate a radiographic response with a pathological response, we next followed a patient (case 3) treated with neoadjuvant osimertinib in a clinical trial (NCT03433469), whose tumor harbored the *EGFR* *L858R* and RBM10 Q595* co-occurring mutations. Following 2 months of neoadjuvant osimertinib treatment, we confirmed low tumor cell RBM10 expression and observed radiographic SD, with 80% tumor cell viability in the osimertinib-treated, resected tumor specimen upon clinical pathology assessment (Figure 4D). In a fourth clinical case, an *EGFR* *L858R* NSCLC harbored a co-occurring *RBM10 c2167-1 G>T* splice site mutation, as detected by clinical-grade NGS. This patient was enrolled in the same neoadjuvant osimertinib clinical trial and also showed a minimal EGFR TKI response, with 68.3% tumor cell viability in the osimertinib-treated, resected tumor specimen upon clinical pathology assessment (Supplemental Figure 8, A and B). Consistent with these clinical data, other investigators observed early clinical progression associated with decreased sensitivity to EGFR TKI therapy in a patient with *EGFR* *L858R* lung cancer whose tumor also harbored a clonal co-occurring truncating *RBM10* mutation (13). Thus, at the individual patient level, across multiple cases, RBM10 inactivation was associated with decreased initial EGFR TKI sensitivity, relatively early clinical progression, and a decreased pathological tumor response in *EGFR*-mutant LA.

To further establish the functional impact of the patient-derived *RBM10* mutations, we engineered the RBM10 Y36*, RBM10 S167*, and RBM10 Q595* variants and induced the expression of each mutant form in RBM10-deficient, *EGFR*-mutant A014 cells (Figure 4E, Supplemental Figure 6, D–F, and Supplemental Figure 9, A and B; the RBM10 splice site variant in the patient in case 4 proved challenging to engineer). In contrast to WT *RBM10*, each *RBM10* mutant failed to rescue the apoptotic phenotype upon osimertinib treatment, as measured by PARP cleavage (Figure 4F). We also confirmed this effect of RBM10 loss on EGFR TKI–induced apoptosis in H3255 and PC-9 *RBM10*-KO cells (Supplemental Figure 10). These findings indicate that the *RBM10* mutations present in the human *EGFR-*mutant lung cancers were loss-of-function mutations and resulted in a decreased apoptotic response to EGFR TKI treatment.

We further investigated whether the RBM10 deficiency was due to low expression of RBM10 or a malfunctioning of the truncated mutant form(s). To test this, we inserted an N-terminal 3X FLAG tag into the RBM10 construct. Using this RBM10 construct, we conducted mutagenesis to generate Y36*, S167*, Q595*, and G840fs* mutants and transfected them into RBM10-deficient A014 cells. The mRNA expression levels of all truncation mutations were lower than those of WT, as confirmed by RT-PCR of FLAG, and the level of G840* was higher than that of the other truncation mutations (Supplemental Figure 9, A, D, and E). The data suggest that the G-patch domain functions in RNA stability and that the whole octamer repeat (OCRE) sequence domain is also necessary for RNA stability. Next, we collected cell lysates from the transfected cells to analyze protein expression by Western blotting. No protein expression was detected upon transfection of each truncation mutation. The Q595* and G840fs* *RBM10*-mutant transfected cells show restored expression under treatment with the proteasome inhibitor bortezomib. The findings suggest that the second RRM domain regulated protein translation, because the Y36* and S167* *RBM10* mutants showed no protein expression, even under treatment with the proteasome inhibitor, and a part of the OCRE or G-patch domain was essential for protein stability (Supplemental Figure 9F). From these findings, we conclude that the low mRNA and protein expression of mutant *RBM10* was a key feature underlying the RBM10 deficiency and loss-of-function phenotypes.

In summary, these clinical findings mirror the effects of RBM10 deficiency that we observed in in vitro and in vivo in the preclinical models, namely diminished initial tumor cell apoptosis and EGFR TKI response, and complement the independent clinical data described above (Figure 4A and Table 3). While larger clinicogenomic cohorts of *EGFR*-mutant tumors treated with an EGFR TKI and data on their treatment outcome status are lacking, our collective findings provide proof of concept of a role for RBM10 deficiency in limiting the EGFR TKI response and offer a rationale for further analysis as additional clinical patient data become available in the future.

### RBM10 deficiency decreases the Bcl-xS to Bcl-xL ratio to limit the apoptotic response to EGFR TKI therapy.

Our studies suggested that loss of *RBM10* could limit EGFR TKI–induced apoptosis rather than increase the proliferative capacity of cancer cells. To investigate the mechanism by which RBM10 deficiency decreases treatment-induced apoptosis, we first undertook a global analysis to identify which mRNAs were alternatively spliced in response to RBM10 status using an established, independent data set (37). This data set was derived from an analysis of RBM10-replete or -deficient HEK293 cells. The analysis revealed 412 genes whose differential mRNA splicing was regulated by *RBM10* KD (Supplemental Figure 11A and Supplemental Table 3) (37). Biological pathway analysis of these 412 targets using established databases (38–40) revealed a significant enrichment for “cell death pathways” with high statistical significance (*P <* 0.05; second highest rank) (Supplemental Figure 11A). We focused on cell death regulation downstream of RBM10, because we noted that RBM10 deficiency suppressed apoptosis during EGFR TKI treatment in the preclinical models (as shown above).

Among these 412 genes, we sought to identify the specific apoptosis-related genes through which RBM10 could function to limit the EGFR TKI response. RBM10 is known to regulate mRNA splicing of factors, such as Bcl-x and caspase 9, involved in intrinsic (mitochondria-mediated) apoptosis (29). Bcl-x, a member of the Bcl-2 family of proteins, is a mitochondria-associated protein that regulates apoptosis (18). Bcl-x can generate either of 2 expressed proteins as a result of alternative mRNA splicing: a short proapoptotic form, Bcl-xS, and a longer antiapoptotic form, Bcl-xL (28, 29). We found that *RBM10* KD decreased the Bcl-xS to Bcl-xL ratio to favor a potential antiapoptotic phenotype (Supplemental Figure 11B). This relationship with RBM10 status appeared to be specific to Bcl-x, as we noted no other Bcl-2 family genes whose mRNA splicing was significantly affected by *RBM10* KD in the global HEK293 cell data set (Supplemental Table 3) (37). Expression of the truncated isoform of *Caspase 9*, *Caspase 9b* (also called *Caspase 9s*), which can be regulated by RBM10 in certain contexts and is antiapoptotic, was not increased by *RBM10* KD in this analysis (Supplemental Table 3) (37), again suggesting a specific association between RBM10 status and Bcl-x mRNA splicing. The broader array of mRNA splicing alterations and the overall effect on *Bcl-x* isoform ratio expression regulated by RBM10 were evaluated by isoform analysis of RNA-Seq data derived from PC-9 shScr and sh*RBM10* cells (Supplemental Table 4). This analysis confirmed the *Bcl-x* isoform ratio expression change upon RBM10 deficiency that was observed in the other preclinical models (i.e., a decreased *Bcl-xS/Bcl-xL* ratio upon RBM10 loss) (Supplemental Figure 12 and data above and below in additional systems).

**Figure 5:**
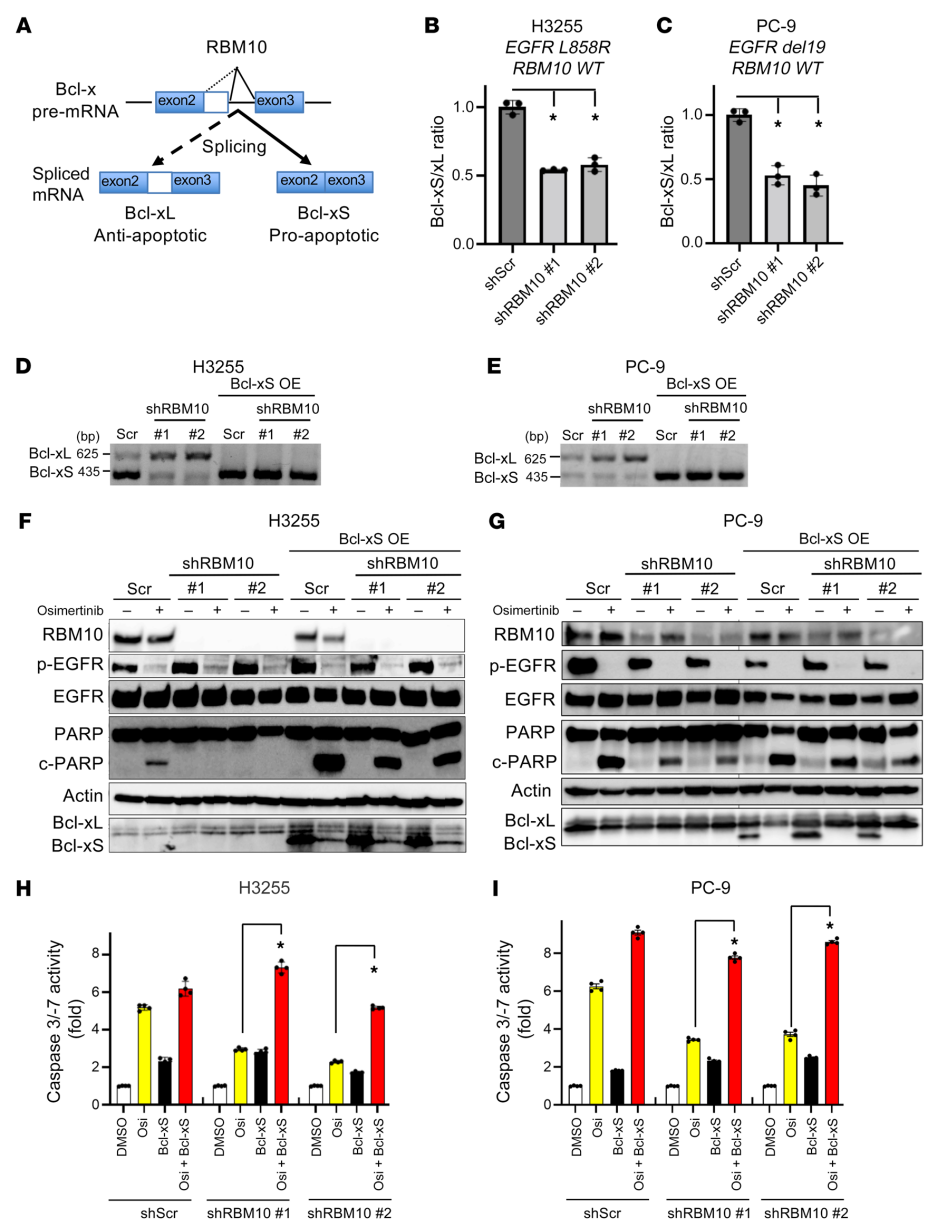
RBM10 deficiency decreases the Bcl-xS to Bcl-xL ratio to limit the apoptotic response to EGFR TKI therapy. (**A**) RBM10 regulates *Bcl-x* mRNA splicing into *Bcl-xS* (proapoptotic) and *Bcl-xL* (antiapoptotic) isoforms. (**B** and **C**) qRT-PCR analysis of the Bcl-xS to Bcl-xL ratio (mRNA levels) in H3255 (**B**) and PC-9 (**C**) cells expressing sh*RBM10* or shScr control. Data are shown as the mean ± SEM of the FC after normalization to the housekeeping gene (*GAPDH*). (**D** and **E**) Conventional PCR analysis using validated primers to detect both Bcl-xL and Bcl-xS isoforms in H3255 and PC-9 cells expressing either shScr control or sh*RBM10* with or without genetic rescue of *Bcl-xS*. (**F** and **G**) H3255 and PC-9 (*EGFR L858R* and *EGFR del19*, respectively; WT *RBM10*) cells treated with osimertinib for 48 and 72 hours, which express either sh*RBM10* or shScr control paired with or without genetic rescue of Bcl-xS. Cell lysates were harvested, and expression of the indicated proteins was measured by Western blotting. (**H** and **I**) Caspase 3/-7 activity was measured using the Caspase-Glo 3/7 assay. Each bar represents the mean ± SEM of the FC after normalization to the DMSO control. Data represent 3 independent experiments. *P < 0.05, by 1-way ANOVA.

Given these collective findings, we focused on *Bcl-x* and hypothesized that RBM10 loss could decrease the Bcl-xS to Bcl-xL ratio, limiting the apoptotic response to EGFR-targeted therapy (Figure 5A). To test this, we performed Bcl-x qRT-PCR analysis in H3255 and PC-9 cells that were either RBM10 replete (shScr) or depleted (*RBM10* KD). We observed a decrease in the Bcl-xS to Bcl-xL ratio, with a relative increase in the antiapoptotic Bcl-xL transcript upon *RBM10* silencing (Figure 5, B–E). The decreased *Bcl-xS* to *Bcl-xL* ratio was also confirmed by qRT-PCR in sg*RBM10* compared with sgControl tumors (Supplemental Figure 4, E and F). We analyzed the correlation between *RBM10* expression and the *Bcl-xS* to *Bcl-xL* ratio in quartiles in a total of 87 *EGFR*-mutated NSCLC clinical samples, which are included in Supplemental Figure 7A. Interestingly, we found that higher mRNA expression of *RBM10* was significantly correlated with a higher *Bcl-xS* to *Bcl-xL* ratio (*P =* 0.0441, ANOVA; Supplemental Figure 7B). A significantly higher *Bcl-xS* to *Bcl-xL* ratio was also observed in WT *RBM10* tumors compared with *RBM10*-mutant tumors (*P =* 0.0045; Supplemental Figure 13). The collective data are consistent with our preclinical findings. The decreased Bcl-xS to Bcl-xL ratio occurred concomitantly with a decreased apoptotic response upon EGFR TKI treatment in H3255 and PC-9 cells (Figure 5, F and G). Genetic rescue of *Bcl-xS* expression in the RBM10-deficient cells restored EGFR TKI–mediated apoptosis to levels comparable to RBM10 replete cells (Figure 5, F–I), providing evidence of the functional relevance of *Bcl-xS* expression in the apoptotic response to EGFR TKI treatment.

**Figure 6:**
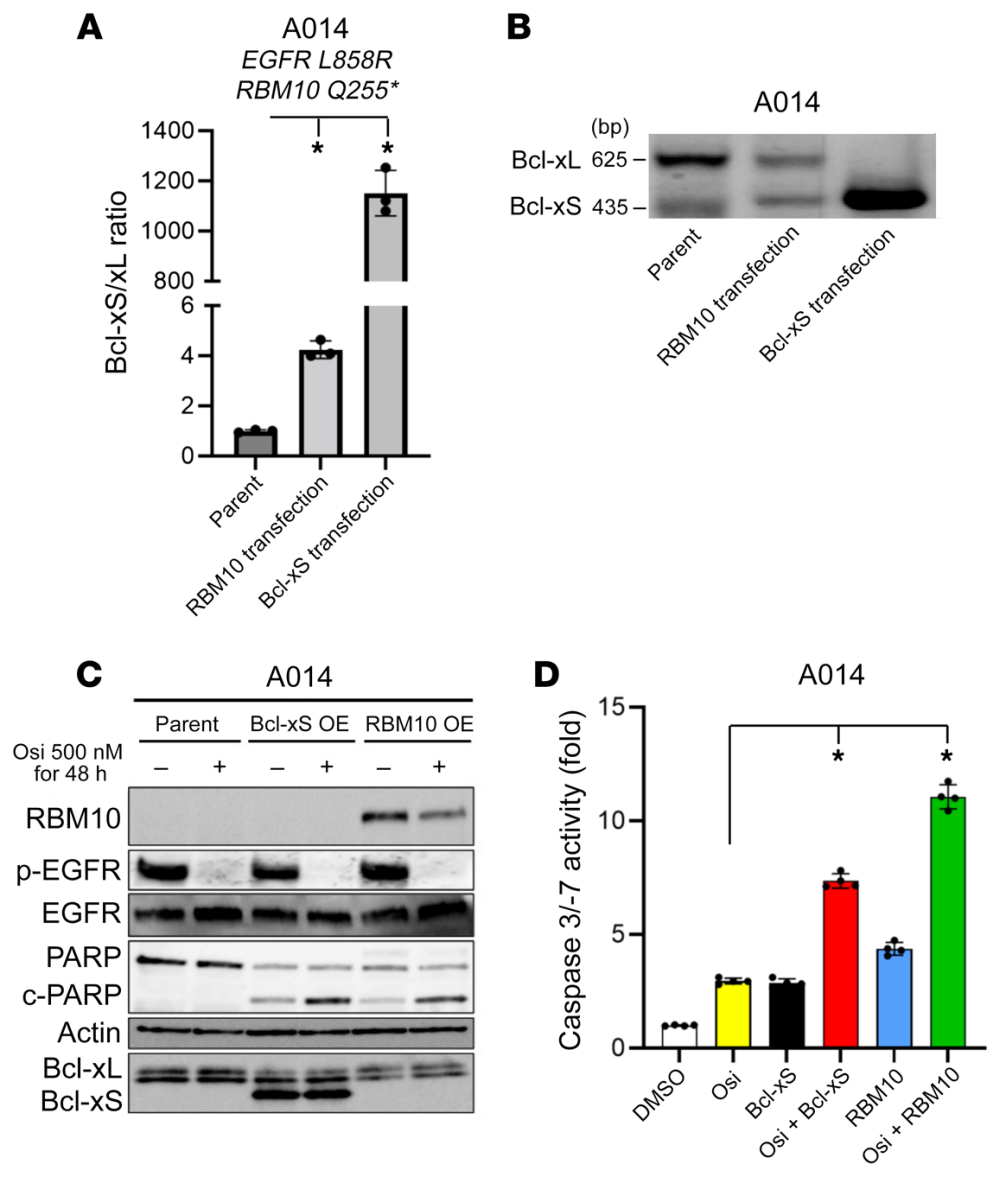
RBM10 transfection recovers the Bcl-xS to Bcl-xL ratio and the apoptotic response to EGFR TKI therapy. (**A**) qRT-PCR and (**B**) conventional RT-PCR analysis of the Bcl-xS to Bcl-xL ratio (mRNA levels) following genetic reconstitution of *RBM10* or *Bcl-xS* in RBM10-deficient A014 cells. (**C**) A014 cells (RBM10-deficient) overexpressing *Bcl-xS* or reconstituted with *RBM10* 24 hours before treatment with osimertinib (500 nM) or the DMSO control for 48 hours. Cell lysates were harvested, and the indicated proteins were measured by Western blotting. (**D**) Caspase 3/-7 activity was measured with the Caspase-Glo 3/7 assay. Each bar represents the mean ± SEM of the FC after normalization to the DMSO control. Data represent 3 independent experiments. *P < 0.05, by 1-way ANOVA.

We next investigated whether exogenous *Bcl-xS* or *RBM10* expression in RBM10-deficient A014 cells could increase the Bcl-xS to Bcl-xL ratio to enhance EGFR inhibitor–mediated apoptosis. Indeed, we observed increased PARP cleavage and caspase 3/-7 activity in A014 cells with genetic reconstitution of *RBM10* or *Bcl-xS* OE when treated with osimertinib (Figure 6, A–D). Furthermore, each of the *RBM10* mutants that were present in the clinical cases shown in Figure 4 failed to increase the Bcl-xS to Bcl-xL ratio, in contrast to WT RBM10 (Supplemental Figure 9B). These findings further corroborate the loss-of-function effect of these *RBM10* variants. We also studied H1975 (*EGFR L858R/T790M*; *RBM10* G840fs*7) cells that are RBM10 deficient at baseline and used a lower osimertinib concentration, because these cells harbor *EGFR T790M*, which may help to confer relatively greater osimertinib sensitivity (41). Similar to A014 cells, reconstitution of *RBM10* or *Bcl-xS* OE in H1975 cells increased the Bcl-xS to Bcl-xL ratio and osimertinib-mediated apoptosis (Supplemental Figure 14, A–G). The collective data indicate that the decreased EGFR inhibitor–mediated apoptosis we observed in RBM10-deficient, *EGFR-*mutant LA was at least in part due to a low Bcl-xS to Bcl-xL ratio.

To further understand the cell-based mechanisms of RBM10/Bcl-xS–mediated apoptosis induction, we assessed mitochondrial membrane potential upon EGFR inhibition, as measured by mitochondrial matrix pH using an established ratiometric pH-sensitive probe, SypHer-dmito (42, 43). We found that *RBM10* KD in PC-9 and H3255 cells resulted in a higher mitochondrial matrix pH (higher SypHer to dmito ratio [F470/F430]), indicative of increased mitochondrial membrane potential and decreased apoptosis induction compared with the shScr control (Supplemental Figure 15, A and B). In contrast, exogenous expression of *RBM10* or *Bcl-xS* in *RBM10*-deficient H1975 and A014 cells lowered the mitochondrial membrane potential compared with control (Supplemental Figure 15, C and D). These findings indicate that RBM10 and Bcl-xS can modulate the mitochondrial apoptotic response to EGFR TKI therapy. While we cannot rule out a role for RBM10-regulated mRNA splicing targets beyond Bcl-x in modulating EGFR TKI sensitivity — an area for future investigation — our data establish an important and, to our knowledge, previously unknown function for differential *BCL-x* mRNA splicing by RBM10 in this context.

### Resistance caused by RBM10 deficiency in EGFR-mutant LA can be overcome with Bcl-xL and EGFR inhibitor combination therapy.

**Figure 7:**
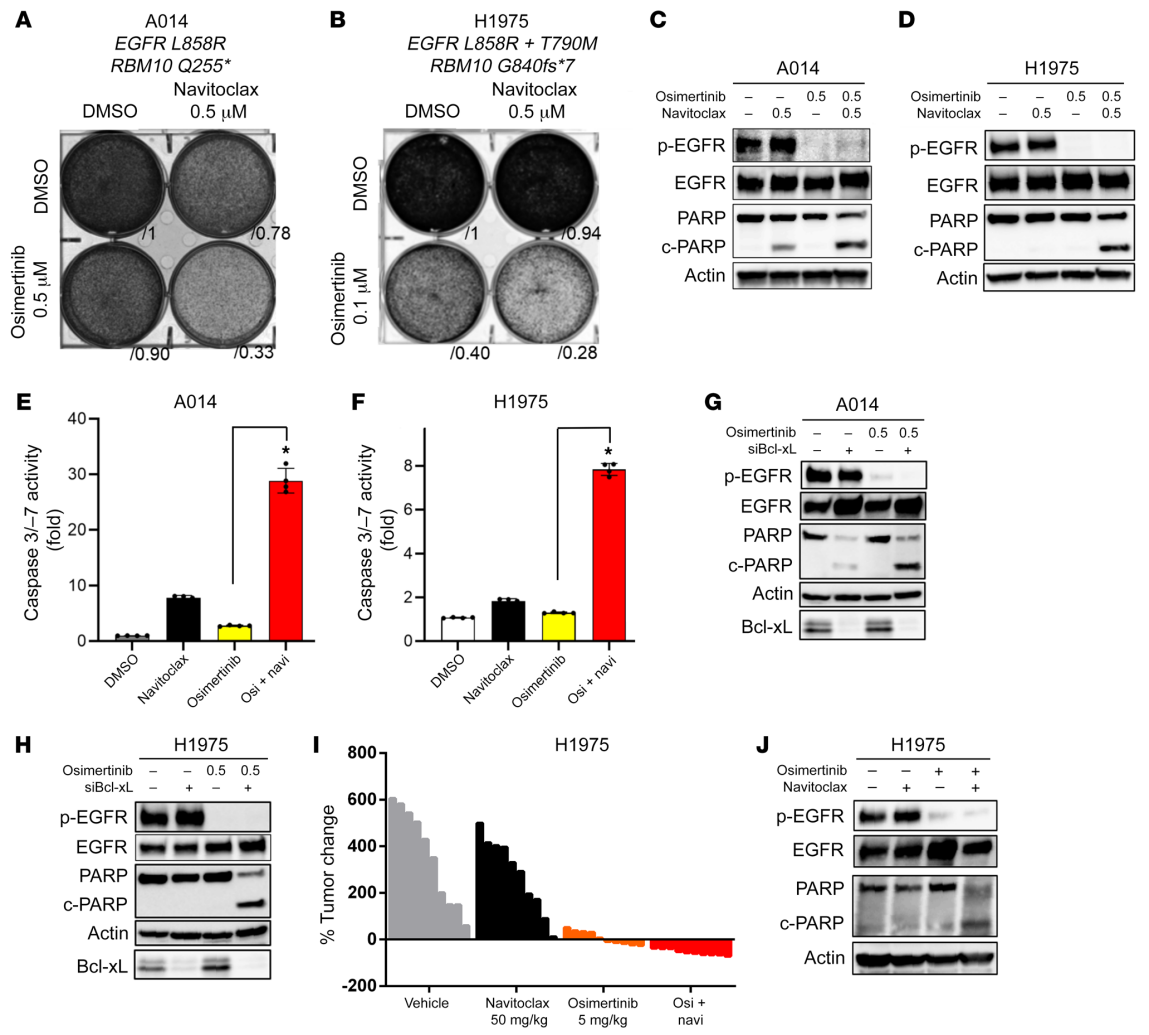
Resistance caused by RBM10 deficiency in *EGFR*-mutant lung cancer can be overcome with Bcl-xL and EGFR inhibitor combination therapy. (**A**–**F**) RBM10-deficient A014 (EGFR L858R; RBM10 Q255*) and H1975 (*EGFR L8585R/T790M*; RBM10 G840fs*7) cells were treated with 500 nM navitoclax (ABT-263) alone or in combination with the indicated osimertinib concentrations. (**A** and **B**) Crystal violet viability assays were performed, and (**C**–**F**) apoptosis was measured according to PARP cleavage and caspase 3/-7 activity. (**E** and **F**) Each bar represents the mean ± SEM of the FC after normalization to the DMSO control. (**G** and **H**) Western blot analysis of *Bcl-xL* KD with siRNA in combination with 500 nM osimertinib in A014 and H1975 cells. (**I**) Mice bearing H1975 subcutaneous xenografts were treated with vehicle, navitoclax (50 mg/kg), osimertinib (5 mg/kg), or their combination (navitoclax plus osimertinib) for 14 days (*n =* 10 tumors in each group). The percentage of change in tumor volume compared with baseline for individual xenografts is shown. (**J**) H1975 xenograft tumor explants were treated with vehicle, navitoclax, osimertinib, or their combination (navitoclax plus osimertinib at 50 mg/kg and 5 mg/kg, respectively) for 4 days. One tumor of representative size from each group was harvested 4 hours after treatment on day 5, and the indicated protein levels were determined by Western blot analysis. Data represent 3 independent experiments. *P < 0.05, by 1-way ANOVA.

The Bcl-xS to Bcl-xL ratio was decreased upon RBM10 loss, resulting in increased Bcl-xL expression relative to Bcl-xS expression. Thus, we investigated whether inhibition of the antiapoptotic isoform of Bcl-x, Bcl-xL, could restore apoptosis in *EGFR*-mutant, *RBM10-*KD cells treated with osimertinib. To test this, we used the BH3 mimetic Bcl-xL inhibitor navitoclax (ABT-263), which binds with high affinity to Bcl-xL (44), in combination with osimertinib and observed decreased viability with enhanced PARP cleavage and caspase 3/-7 activity in 2 independent RBM10-deficient, *EGFR*-mutant cell lines (A014: EGFR L858R; RBM10 Q255* and H1975: EGFR L8585R/T790M; RBM10 G840fs*7) (Supplemental Figure 6, D–G, and Figure 7, A–F). Additionally, navitoclax treatment rescued osimertinib-mediated apoptosis in H3255 and PC-9 cells with RBM10 loss via sg*RBM10*-mediated silencing (Supplemental Figure 16, A and B). Although navitoclax can target other proteins beyond Bcl-xL, including Bcl-2 (44), we used an independent genetic approach to corroborate the role of Bcl-xL by silencing *Bcl-xL* in RBM10-deficient cell lines (A014 and H1975). Under these conditions, we observed enhanced apoptosis in combination with osimertinib (Figure 7, G and H). This benefit from the osimertinib-plus-navitoclax combination in RBM10-deficient tumors, but not RBM10-intact tumors, was confirmed using the isogenic H3255 and PC-9 systems as well (Supplemental Figure 16, C–F). These findings indicate that pharmacologic or genetic suppression of *Bcl-xL* can overcome EGFR inhibitor resistance in RBM10-deficient, *EGFR*-mutant LA cells.

In order to further validate these therapeutic findings in vivo, we treated mice bearing H1975 tumor xenografts with navitoclax, osimertinib, or combination (navitoclax plus osimertinib) therapy (Figure 7, I and J) (A014 cells did not form tumors in vivo). All mice treated with the navitoclax and osimertinib combination achieved an objective response (partial response or complete response by RECIST, version 1.1 criteria; ref. 3), whereas no objective responses were observed in mice treated with either navitoclax or osimertinib alone (Figure 7I and Table 4). Analysis of the tumor explants revealed an increase in PARP cleavage in H1975 tumors obtained from mice treated with the combination of navitoclax and osimertinib (Figure 7J).

Altogether, these findings indicate that RBM10 deficiency suppresses mitochondria-mediated apoptosis in response to EGFR inhibition in *EGFR*-mutant LA by decreasing the Bcl-xS to Bcl-xL ratio. The EGFR TKI insensitivity induced by RBM10 deficiency can potentially be addressed with combination therapies that target the antiapoptotic isoform of Bcl-x, Bcl-xL, together with an EGFR TKI.

## Discussion

This study addresses an emerging and important question: What, if any, is the impact of co-occurring genetic alterations in cancers harboring a canonical primary driver mutation, such as mutant *EGFR*? Our data highlight the increasing need to delineate the genetic heterogeneity present both within and between *EGFR*-mutant tumors and to understand the functional consequences of this genetic heterogeneity to improve clinical outcomes. Our findings provide insight by revealing a previously unappreciated role for co-occurring *RBM10* deficiency in limiting the initial response to EGFR inhibitor treatment in human *EGFR*-mutant LA by suppressing tumor cell apoptosis. The role of RBM10 deficiency in limiting tumor cell apoptosis during this early period of initial therapy is distinct from mechanisms that promote the subsequent emergence of acquired resistance after an initial robust tumor response, such as *EGFR T790M* or *MET* kinase amplification, which often drives tumor cell proliferation (8–13). Our data suggest 1 model for the multifaceted evolution of resistance, such that RBM10 inactivation, for instance via subclonal mutation, may allow for a fraction of cancer cells to avoid apoptosis and persist during initial targeted therapy, resulting in an incomplete tumor response. This is consistent with our observations that tumors with RBM10 deficiency generally were not completely intrinsically resistant and instead showed a suboptimal response, while persisting during EGFR TKI treatment. Subsequent frank tumor progression may then occur via the acquisition of other genetic events that further drive acquired resistance, such as drug-resistant secondary mutations in the *EGFR* or the activation of bypass signaling pathways. We detected the *EGFR T790M* mutation that causes resistance to first-generation EGFR TKIs in the erlotinib-resistant PC9sg*RBM10* and H3255sg*RBM10* cells after 2 months of treatment in vitro. Interestingly, the *T790M* sequence peaks appeared greater in the RBM10-deficient resistant cells than in the RBM10-proficient resistant cells (Supplemental Figure 17), suggesting that RBM10 loss could be conducive to the emergence of certain known resistance mechanisms over time. Thus, RBM10 deficiency may function distinctly from, yet cooperate with, additional molecular events to limit the tumor response to EGFR TKI treatment and enable drug-resistant tumor progression that is, over time, lethal in patients.

Although other reports indicated recurrent *RBM10* mutations in genetically unselected LA patients, the relatively low frequency of *EGFR*-mutant tumors in these published data sets precluded a subtype-specific analysis (25, 26, 31). *RBM10*-truncating mutations were more frequently observed in the *EGFR L858R* subtype compared with tumors that harbored *EGFR* exon 19 deletions (15% versus 3%, *P <* 0.01; Figure 1B). Given the functional role of RBM10 loss in limiting therapeutic responses to EGFR inhibitors, our data reveal a potential mechanism that helps explain why patients with *EGFR L858R* mutation tumors generally have worse clinical outcomes and decreased EGFR inhibitor responses compared with patients with *EGFR* exon 19 deletion tumors (45). How or why *RBM10* genetic alterations are enriched in the *EGFR L858R*–mutant subtype remains unexplained and will be the focus of future studies. An additional area of investigation includes the role of RBM10 deficiency in lung cancer pathogenesis in the absence of systemic therapy, given the differential findings observed in this treatment-naive context in the human systems in our study versus recent data in murine models (46).

Our study sheds light on the role of alterations in mRNA splicing factors in cancer pathogenesis. Somatic mutations in genes encoding the spliceosome have been identified in hematopoietic malignancies, including in up to 60% of patients with myelodysplastic syndrome (MDS). These mutations commonly occur in splicing factor 3b subunit 1 (*SF3B1*), serine/arginine-rich splicing factor 2 (*SRSF2*), and U2 small nuclear RNA auxiliary factor 1 (*U2AF1*), and the genetic data in MDS suggest that these alterations are critical to disease pathogenesis (23). Yet, only some of the mutations in the splicing regulators that are recurrently altered in hematopoietic malignancies have been detected in solid tumors to date (24).

Our findings provide an initial example, to our knowledge, of a mechanistic role for splicing factor inactivation (here, RBM10 deficiency) in modulating sensitivity to targeted kinase inhibitor therapy in solid malignancies. RBM10 deficiency does not modulate the oncoprotein target itself (here, mutant EGFR), but instead functions via the differential regulation of the apoptotic machinery in tumor cells. In contrast, truncated forms of mutant BRAF are associated with resistance to BRAF inhibitor treatment as a form of “on-target” therapy resistance in melanoma and lung cancer (47, 48). Whether these truncated mutant BRAF forms arise via alternative mRNA splicing and, if so, the precise splicing factor involved are unresolved questions. Thus, splicing factor deficiency (here, of RBM10) per se appears to represent a distinct mechanism of targeted kinase inhibitor therapy resistance, as reported in previous studies (8–13).

Our findings indicate that *RBM10* deficiency in *EGFR*-mutant LA tumors decreased the apoptotic response to EGFR inhibitor therapy, leading to tumor progression during EGFR TKI treatment and worse clinical outcomes. RBM10 controls alternative splicing of the apoptosis regulator Bcl-x to generate 2 isoforms: Bcl-xL (antiapoptotic) and Bcl-xS (proapoptotic) (28, 29). Bcl-x is a member of the Bcl-2 family of proteins that exist at the outer mitochondrial membrane. Bcl-2 family proteins regulate mitochondrial outer membrane permeabilization and the release of cytochrome c into the cytoplasm in response to EGFR TKI treatment (49). An alternative splicing event in exon 2 of *Bcl-x* results in 2 isoforms of *Bcl-x* with antagonistic effects on cell survival: Bcl-xL (long isoform), which is antiapoptotic, and Bcl-xS (short isoform), which is proapoptotic (49). Mechanistically, RBM10 deficiency alters *Bcl-x* splicing to increase the relative abundance of its antiapoptotic isoform, Bcl-xL, to limit apoptosis upon EGFR TKI therapy. This antiapoptotic molecular effect arising in RBM10-deficient cells can be overcome by Bcl-xL inhibition (pharmacologic or genetic). Our findings indicate that the mutation and/or expression status of *RBM10* could be a promising biomarker of response to the combination of osimertinib and navitoclax in a clinical trial (50), an area for future investigation that could serve to refine patient selection for treatment and improve clinical outcomes. Beyond Bcl-x, RBM10 may regulate, via mRNA alternative splicing, other genes that are involved in therapeutic responses, yet another avenue for future study.

In summary, our findings illustrate the utility of understanding the role of co-occurring genetic alterations in oncogene-driven cancers, with translational implications. The effect of RBM10 deficiency on *EGFR*-mutant NSCLC established in this study sheds light on the role of tumor genetic heterogeneity in the multifaceted evolution of therapeutic resistance.

## Methods

### Global analysis of alternative mRNA splicing regulated by RBM10 in HEK293 cells

Splicing changes induced by *RBM10* KD (si*RBM10* KD) in HEK293 cells were based on RNA-Seq data (37). Briefly, the inclusion ratio (percentage splicing in [PSI]) of each exon in RefSeq transcripts was the number of reads supporting inclusion divided by the total number of reads supporting inclusion and exclusion of the specific exon. The inclusion ratio between RBM10 and the control was computed and then transformed into a *z* value (37). Functional annotation of these differentially spliced genes was carried out using multiple databases (Kyoto Encyclopedia of Genes and Genomes [KEGG], Biological Biochemical Image Database [BBID], and BioCarta) (38–40). The adjusted *P* value cutoff for significant gene sets was set at 0.05. A hypergeometric test was used for pathway enrichment analysis within an algorithm (Database for Annotation, Visualization, and Integrated Discovery [DAVID] 6.8) (38–40).

### Analysis of RBM10-mediated differential alternative mRNA isoforms in PC-9 lung cancer cells

Total RNA was isolated from PC-9 parental and PC-9 cells with *RBM10* KD (sh*RBM10*) and subjected to transcriptome sequencing. The alternative isoform RNA transcripts were quantified using the RSEM algorithm, and the differential expression of isoform RNA transcripts between the *RBM10*-KD and control groups were defined using the EdgeR package in R. The differential mRNA isoform expression criteria were set as a |log_2_ fold change (FC)| of greater than 1 and a FDR of less than 0.05. The RNA-Seq data have been deposited in the NCBI’s Gene Expression Omnibus (GEO) database (GEO GSE199240).

### Cell lines and culture reagents

The A014 cell line was provided by Daniel Tan (Cancer Therapeutics Research Laboratory, Division of Medical Sciences, National Cancer Centre, Singapore). Whole-exome sequences were deposited in the NCBI’s Sequencing Read Archive (SRA) under the accession code PRJNA816272. PC-9, H3255, and H1975 cells were purchased from the American Type Culture Collection (ATCC). Cells were maintained at 37°C in a humidified atmosphere at 5% CO_2_ and grown in RPMI medium 1640 supplemented with 10% (v/v percentage) FBS, 100 IU/mL penicillin, and 100 μg/mL streptomycin.

### Cell viability assay

For Crystal violet experiments, 3 × 10^5^ cells were plated in 6-well adherent dishes (Corning). After 24 hours, cells were exposed to either vehicle (DMSO), osimertinib, or navitoclax. Each assay was performed in triplicate, and representative images are shown. Densitometric quantifications were performed with ImageJ software (NIH).

### Cell apoptosis

Cells (3 × 10^3^) were seeded in 96-well, white-walled plates (Corning) and incubated overnight. Cells were subsequently treated with vehicle (DMSO) or the indicated compounds for 48 hours. Cellular apoptosis was analyzed with Caspase-Glo 3/7 assay kits (Promega), which measures caspase 3/-7 activity, in accordance with the manufacturer’s protocol.

### Western blot analysis

Cells (2 × 10^5^) were seeded in 6-well plates and rested overnight before drug treatment for 48–72 hours. Whole-cell lysates were prepared using RIPA (10 mM Tris-Cl [pH 8.0], 1 mM EDTA, 0.1% sodium deoxycholate, 0.1% SDS, 140 mM NaCl) supplemented with a protease inhibitor and a phosphatase inhibitor (both from Roche) and clarified by probe sonication and centrifugation (14,000*g* for 15 minutes at 4°C). Equal masses of protein (5 μg–40 μg) were separated by 4%–15% of SDS/PAGE and transferred onto nitrocellulose membranes (Bio-Rad) for protein blot analysis. Membranes were incubated with a primary antibody overnight and washed and incubated with a secondary antibody for 1 hour. Protein bands were visualized using either a fluorescence system (LI-COR) or Amersham ECL chemiluminescent reagent (GE Life Sciences); chemiluminescent signals were visualized with an ImageQuant LAS 4000 instrument (GE Healthcare).

### Antibodies

Antibodies against phosphorylated EGFR (p-EGFR) (Tyr1068) (catalog 3777); total-EGFR (catalog 54359); p-ERK (catalog 4370); total-ERK (catalog 4695); β-actin (catalog 3700); Bcl-xL (catalog 2746); and cleaved PARP (catalog 9546) were purchased from Cell Signaling Technology and diluted according to the manufacturer’s recommendations. The RBM10 antibody (catalog sc-515548) used for Western blotting was purchased from Santa Cruz Biotechnology and diluted.

### Time-lapse imaging

Cells (5 × 10^4^) were seeded in a 35 mm petri dish containing a 14 mm Microwell No. 1.5 coverglasss (0.16–0.19 mm, MatTek) for adherent live cell imaging. The cells stably expressed the SypHer-dmito construct (42). Forty-eight hours before image acquisition, the cells were transfected with either the *RBM10* or *Bcl-xS* constructs for genetic OE experiments or the si*RBM10* variants for genetic KD experiments. Immediately before imaging, media were replaced with physiological salt solution (PSS) containing 150 mM NaCl, 4 mM KCl, 2 mM CaCl_2_, 1 mM MgCl_2_, 5.6 mM glucose, and 25 mM HEPES (pH 7.4) with or without 50 mM dichloroacetic acid (DCA) at 37°C. Images were acquired with a CMOS image sensor, ORCA-Flash 4.0 (Hamamatsu), equipped with 418–442 nm and 450–490 nm excitation filters and a 510–540 nm emission filter. The SypHer-dmito fluorescence (F470/F430) ratio was calculated for each cell after subtracting the background signal. All images were analyzed with ImageJ software (43). For each cell type, cells with F470/F430 ratios of greater than 2 standard deviations from the mean value were excluded.

### GeneKD and OE assays

All shRNAs for *RBM10* were obtained from MilliporeSigma (TRCN0000233276 and 0000233277). Sequences for the individual shRNAs were as follows: shRBM10 no. 1, TRCN0000233276, CCGGGACATGGACTACCGTTCATATCTCGAGATATGAACGGTAGTCCATGTCTTTTTG; shRBM10 no. 2, TRCN0000233277, CCGGCTTCGCCTTCGTCGAGTTTAGCTCGAGCTAAACTCGACGAAGGCGAAGTTTTTG.

Stealth RNAi (Dharmacon) Bcl-xL and the Stealth RNAi-negative control were used for RNAi assays as described in the manufacturer’s guide. The siRNA target sequences for Bcl-xL were as follows: 5′-CUCCUUCGGCGGGGCACUGUGUU-3′ and 5′-CACAGUGCCCCGCCGAAGGAGUU-3′.

For the derivation of clonal populations and generation of *RBM10*-KO cells, clonal cells were derived by sorting single cells into 96-well plates and expanding them over a period of several weeks. We then derived pools of one of the clones expressing either a nontargeting guide or a *RBM10*-targeting guide along and puromycin marker and CRISPR/Cas9 by lentiviral transduction as done in a previously published study (51). A couple of gRNA target sequences (shown below), which were designed by the Zhang laboratory (51) to specifically target *RBM10*, were first subcloned into the all-in-one lentiCRISPR v2 plasmid (GenScript). They were then lentivirally transduced into PC-9 and H3255 clonal cells and tested, and the one that showed better RBM10 depletion in Western blotting was selected for further analysis. Additionally, *RBM10*-KO clones were also generated from clonal derivatives of PC-9 and H3255 cells. However, H3255 was difficult to grow in a single cell, so bulk cells were used. the sequences for the individual sgRNAs were as follows: sg*RBM10* no. 1, 5′-CGTTCATATCCTCGCGAGTA-3′; sg*RBM10* no. 2, 5′-GCACGCCGTGCGACTGCAGC-3′.

### In vivo compound formulation

Osimertinib and navitoclax were obtained from Selleck Chemicals and Chemgood, respectively. For all studies in mice, osimertinib and navitoclax were administered daily by oral gavage. Osimertinib was dissolved in a v/v percentage mixture of 7% DMSO, 13% Tween-80, and 80% 5% glucose, followed by an acid adjustment using an equimolar volume of HCl. Navitoclax was dissolved in 30% PEG 400 (MilliporeSigma), 60% Phosal 50 PG (MilliporeSigma), and 10% ethanol (MilliporeSigma) and vortexed continuously throughout the dosing period.

### Immunostaining

#### Clinical samples and subcutaneous xenografts.

For subcutaneous xenografts studies, mice were sacrificed at the primary endpoint. Tumors were harvested and fixed in 10% neutral buffered formalin for 48 hours, embedded in paraffin, and sections of 5 to 10 μm thickness were prepared. The sections were subsequently deparaffinized in xylene, rehydrated in a graded ethanol series, and pressure boiled with 1× target retrieval buffer, and sodium citrate, pH 6.1 (Agilent Dako) for 1 hour. Tissues were treated with 0.3% H_2_O_2_ for 10 minutes, washed with PBS, and incubated with antibodies directed against RBM10 (A301-006A, Bethyl Laboratories), Ki-67 (catalog 9449, Cell Signaling Technology), and cleaved caspase 3 (catalog 9664, Cell Signaling Technology), and a TUNEL assay kit (Abcam) was used according to the manufacturers’ recommendations. IHC was evaluated by a semiquantitative approach to assign an H score (mean ± SEM).

### Plasmid transfections

Bcl-xS and RBM10 were obtained from Addgene. Plasmid transfections required 3 μg/well and were carried out using 0.1% FuGENE HD (Promega).

### PCR experiments

PCR was performed at 95°C for 15 seconds, followed by 27–30 cycles at 95°C for 15 seconds, 58.5°C for 15 seconds, and 72°C for 20 seconds, and finally at 72 °C for 7 minutes using AmpliTaq Gold PCR Master Mix (Applied Biosystems) with template cDNA equivalent to 15 ng total RNA and a high concentration (0.75 mM each) of primers. The PCR conditions were semiquantitative, and no more than 2%–5% of input primers were consumed. The primer set for *Bcl-x* simultaneously amplified 2 alternatively spliced isoforms (*Bcl-xL*; 625 bp, *Bcl-xS*; 435 bp). The following sequences were used: *Bcl-x* forward, 5′-AGCTGGAGTCAGTTTAGTGATGTG-3′; *Bcl-x* reverse, 5′-TGAAGAGTGAGCCCAGCAGAAC-3′.

### qRT-PCR assay for gene expression

Cells (3 × 10^5^) were seeded and given 24 hours to adhere at 50% confluence. Cells were then treated with inhibitors for 24 hours, followed by rapid RNA extraction using the RNeasy Kit (QIAGEN). cDNA was prepared from 500 ng total RNA with the SensiFAST cDNA Synthesis Kit (Bioline). qPCR was performed with the QuantStudio 12K Flex Real-Time qPCR system (Applied Biosystems) using TaqMan probes (human RBM10: assay ID, Hs00275935_m1; human Bcl-xL: assay ID, Hs00236329_m1; human Bcl-xS: assay ID, Hs00169141_m1; Applied Biosystems). GAPDH expression was used as an internal control to normalize input cDNA (human GAPDH: assay ID, Hs02758991_g1; Applied Biosystems). The ratios of the expression level of each gene to that of the reference gene were then calculated.

#### RBM10 site-directed mutagenesis.

The QuikChange II Mutagenesis Kit (Agilent Technologies) was used to generate all RBM10 mutants. The QuikChange II primer design website was used to guide the generation of mutagenesis primers for the investigated mutants. For transient transfection experiments, FuGENE 6 transfection reagent (Promega) was used according to the manufacturer’s protocols. Primer sequences for the individual mutants were as follows: RBM10 Y36*, left, 5′-CGCTATGGAGCCACTFACC-3′, right, 5′-TACTCGCGAGGATATGAACG-3′; RBM10 S167*, left, 5′-GTGCAAGCACGGGAGGTT-3′, right, 5′-GCTTCCATCCATCGTGTAGC-3′; RBM10 Q595*, left, 5′-GGCTACTACTATGACCCCCAGA-3′, right, 5′-CCTCTCCCCATCCCAGTACA-3′; and RBM10 G840fs*7, left, 5′-AGAGCCCAAGAGGAGGAAGT-3′, right, 5′-CATCCGACTGCCAATGTTGT-3′.

#### Orthotopic lung xenografts in immunodeficient mice.

Six- to 8-week-old female SCID CB.17 mice were purchased from Taconic. Specific pathogen–free conditions and facilities were approved by the Association for Assessment and Accreditation of Laboratory Animal Care International (AAALAC). To prepare cell suspensions for thoracic injection, cells were mixed with Matrigel matrix (BD Bioscience 356237) on ice, yielding a final concentration of 1.0 × 10^5^ cells/μL. Mice were placed in the right lateral decubitus position and anesthetized with 2.5% inhaled isoflurane. A 1 cm surgical incision was made along the posterior medial line of the left thorax, fascia, and adipose tissue layers were dissected and retracted to expose the lateral ribs, intercostal space, and left lung parenchyma. A 30 gauge hypodermic needle was used to advance through the intercostal space approximately 3 mm into the lung tissue. Cells were taken to inject 10 μL (1.0 × 10^6^ cells) cell suspension directly into the left lung. Visorb 4/0 polyglycolic acid sutures were used for closure of the fascia and skin layer. Mice were observed after the procedure for 1–2 hours. For drug treatments, orthotopically implanted tumors were allowed to grow for 1 week before treatment. Mice were treated with either vehicle or osimertinib at the start of week 2 and continued on therapy until week 9 (post-implantation day 60).

#### Subcutaneous tumor xenografts.

Beas2B, H1975, H3255, and PC-9 tumor xenografts were generated by injection of 1 × 10^6^ cells in a 50/50 mixture for Matrigel and PBS into 6- to 8-week-old female NOD/SCID mice. The sg*RBM10* used sg*RBM10* no. 1 for H3255 and sgRBM10 no. 2 for PC-9 in Supplemental Figures 4 and 16. Mice were randomized to treatment groups once tumors reached an average size of 150 mm^3^. For drug treatments, H1975, H3255, and PC-9 cells were subcutaneously implanted and allowed to grow to approximately 200 mm^3^ in size (4 weeks after implantation). Mice were then treated with vehicle, osimertinib, and/or navitoclax for 15 days.

#### In vivo bioluminescence imaging.

Mice were imaged at the UCSF Preclinical Therapeutics Core after tumor injection on day 7 with a Xenogen IVIS 100 bioluminescence imaging system. Before imaging, mice were anesthetized with isoflurane, and 200 μL d-luciferin at a dose of 150 mg/kg body weight was administered by intraperitoneal injection. Weekly monitoring of bioluminescence of the engrafted lung tumors was performed until week 9. Radiance was calculated automatically using Living Image Software following demarcation of the thoracic cavity (region of interest [ROI]) in mice in the supine position. The radiance unit of photons per s^−1^/cm^2^·sr^−1^ is the number of photons per second that leave a square centimeter of tissue and radiate into a solid angle of 1 steradian (sr).

### Statistics

One-way ANOVA or Student’s *t* test was used to calculate *P* values for comparisons of 3 or more groups or 2 groups, respectively. Fisher’s exact test was used to compare the response rate between 2 groups. Wilcoxon’s test was used to compare the Kaplan-Meier curves. All statistical analyses were conducted using JMP8 software (SAS Institute), with a *P* value of less than 0.05 considered statistically significant.

#### LA data set.

Targeted sequencing of lung cancer samples using a panel of 324 cancer-related genes was provided by Foundation Medicine (https://www.foundationmedicine.com/genomic-testing/foundation-one-cdx). The MSK-IMPACT data set was download from cBioPortal (https://www.cbioportal.org/study/summary?id=msk_impact_2017).

TCGA-LUAD RNA-Seq and whole-exome sequencing data were obtained from the genomic data commons (https://gdc.cancer.gov/).

All data were processed using R programming (version 3.6.2). The oncoprint for *EGFR-RBM10* comutations was generated using the ComplexHeatMap package in R.

### Study approval

#### Human EGFR TKI–treated deidentified patient cohort.

Eighty-eight patients with NSCLC with *EGFR* mutations at the Catalan Institute of Oncology, Hospital Germans Trias i Pujol (Badalona, Barcelona, Spain) were treated with EGFR TKIs (see Supplemental Figure 7). Ethics Committee for Research in Medicine (CEIM) of the Quirónsalud-Catalunya Hospital Group (RD 1090) was granted by the IRB on December 4, 2015. Twelve patients at the National Cancer Centre Singapore harboring *EGFR* mutations participated in this study (see Figure 4, A and B). Patients from Singapore were enrolled in studies approved by the SingHealth Centralized Institutional Review Board (CIRB) under protocols of the National Lung Cancer Research (NLCR) (CIRB ref. no. 018/2963) and the Individualized Molecular Profiling for Allocation to Clinical Trials (IMPACT) Project (CIRB ref. no. 2019/2170). Fifteen patients at Kanazawa University carrying *EGFR* mutations participated in this study (see Figure 4, A and B). This study was approved by the relevant IRB of Kanazawa University in Japan (IRB no. 2018-014). Informed consent for the analysis was obtained from all patients All patients’ tumor samples analyzed were obtained under IRB-approved protocols, with informed consent provided by each patient under the guidance of the UCSF. All relevant ethics regulations were followed.

#### UCSF deidentified clinical cases.

IRB approval for study no. 13-12492 was granted by the IRB of the UCSF. According to the federal regulations summarized in 45 CFR 46.102(f), this study did not involve human subjects and thus did not require further IRB oversight. The requirement for informed consent was waived. A retrospective chart review of patients was carried out by the study investigators to identify patients’ demographic information, including objective responses, PFS, and OS following EGFR TKI therapy (Figure 4, A–E, Supplemental Figure 7, and Supplemental Figure 13). Direct radiographic review was performed by the study investigators when possible.

#### Subcutaneous tumor xenograft studies.

All animal experiments were conducted under UCSF IACUC-approved animal protocol no. AN107889-03C. Beas2B, H1975, H3255, and PC-9 tumor xenografts were generated by injection of 1 × 10^6^ cells in a 50/50 mixture for Matrigel and PBS into 6- to 8-week-old female NOD/SCID mice. The sg*RBM10* used sg*RBM10* no. 1 for H3255 and sgRBM10 no. 2 for PC-9 in Supplemental Figures 4 and 16. Mice were randomized to treatment groups once tumors reached an average size of 150 mm^3^. For drug treatments, H1975, H3255, and PC-9 cells were subcutaneously implanted and allowed to grow to approximately 200 mm^3^ in size (4 weeks after implantation). Mice were then treated with vehicle, osimertinib, and/or navitoclax for 15 days.

## Author contributions

RAO and TGB conceived and designed the study. Development of methodology: SN, WW, and RO. SN, WW, NK, CMB, YTC, DLK, VO, J Shue, JR, AU, JCY, AJS, ACT, WLT, OA, KH, and AN acquired data. SN, WW, NK, and J Suzuki analyzed and interpreted data. SN, WW, RAO, and TGB wrote, reviewed, and revised the manuscript. NK, CMB, SMA, MM, FH, NC, DSWT, SY, YK, and RAO provided administrative, technical, and material support. RR, RAO, and TGB supervised the study.

## Supplementary Material

Supplemental data

Supplemental table 3

Supplemental table 4

## Figures and Tables

**Table 3:**
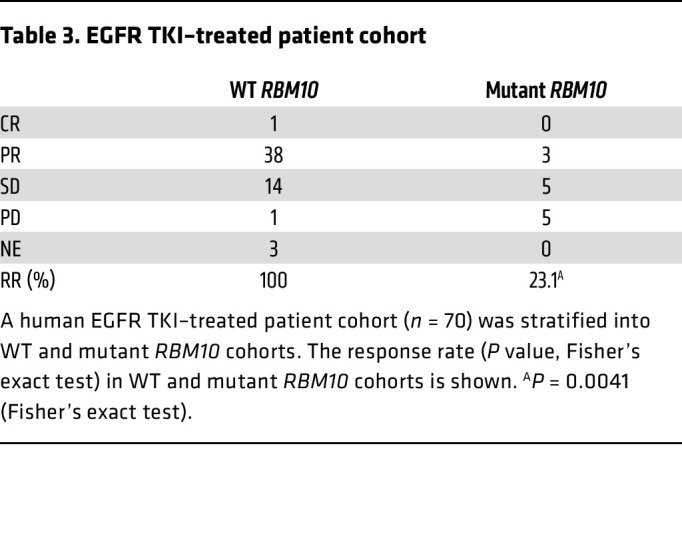
EGFR TKI–treated patient cohort

**Table 2:**
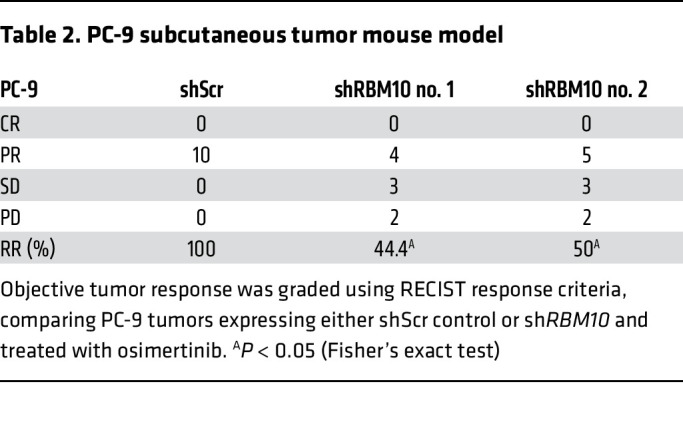
PC-9 subcutaneous tumor mouse model

**Table 1:**
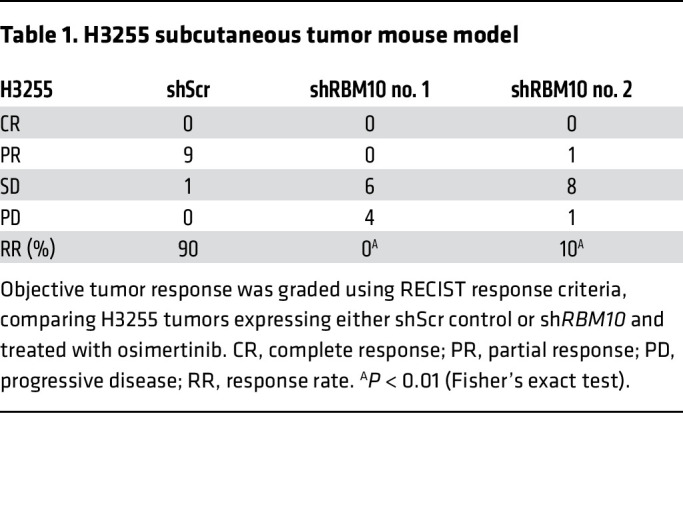
H3255 subcutaneous tumor mouse model

**Table 4:**
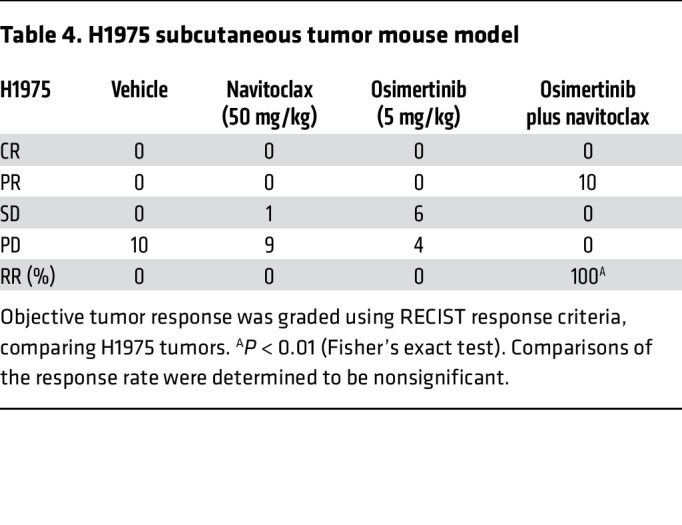
H1975 subcutaneous tumor mouse model

## Version 1 05/17/2022

In-Press Preview

## Version 2 07/01/2022

Electronic publication
