# Supplementary material for: Deficiency of the splicing factor RBM10 limits EGFR inhibitor response in EGFR-mutant lung cancer
Source: J Clin Invest. 2022 Jul 1;132(13):e145099. doi: 10.1172/JCI145099 (PMC9246391; doi:10.1172/JCI145099)
Supplement: Supplemental data [file jci-132-145099-s142.pdf]

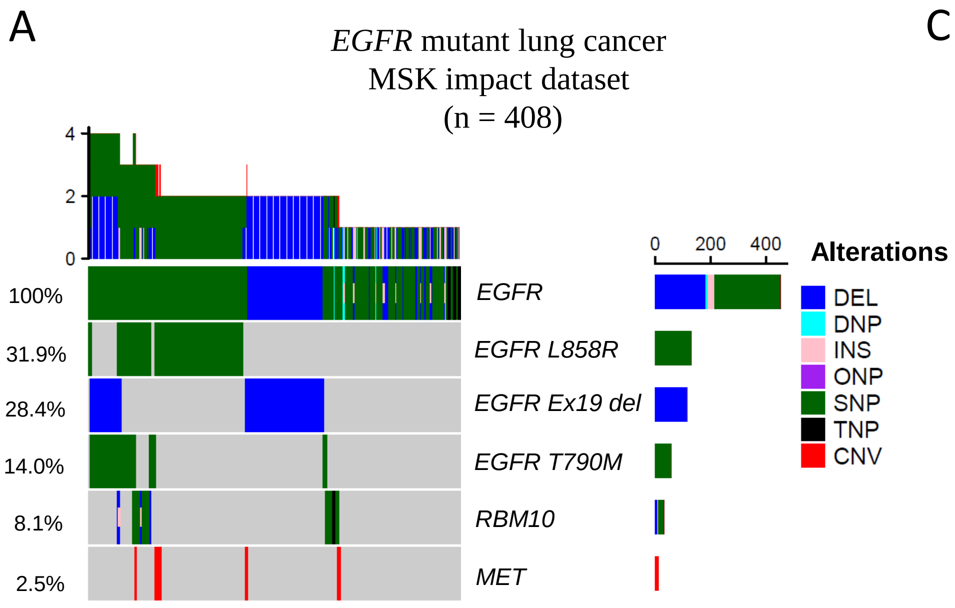

C

RBM10 and *EGFR* mutation clonality estimation
Foundation Medicine dataset

	***EGFR* mutant**	***RBM10* mutant**
clonal	14	4
subclonal	31	41

p = 0.02 (Fisher exact test)

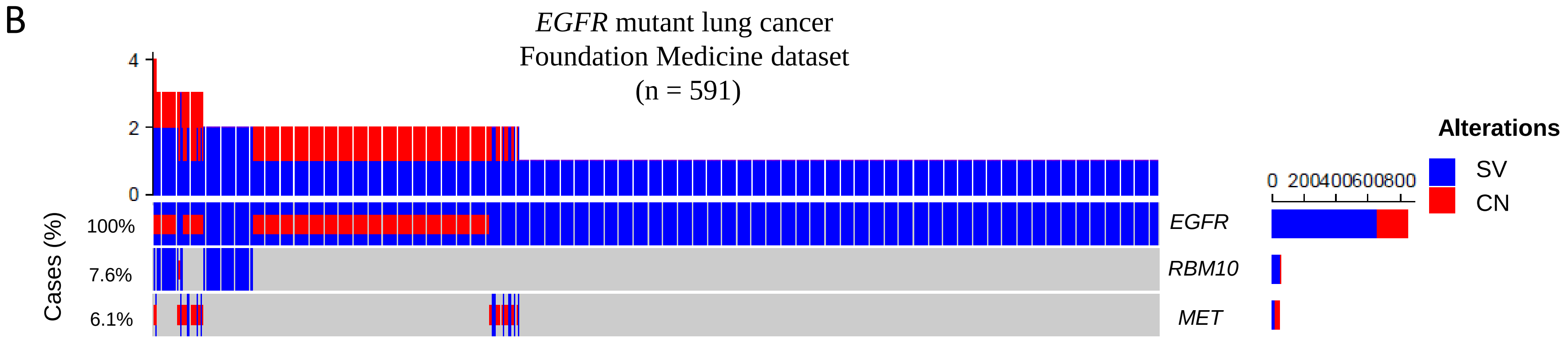

Supplementary Figure 1. *RBM10* mutations and *MET* gene amplification in MSK-IMAPCT and Foundation Medicine *EGFR* mutant lung adenocarcinoma datasets. A, Oncoprint of *EGFR* and *EGFR* allelic mutations along with *RBM10* and *MET* alterations in NSCLC. Targeted deep sequencing data from MSK-IMPACT was used to present the concurrent gene alterations in NSCLC. Different colors represent different genetic alterations: DEL=deletion; DNP=Double nucleotide polymorphism; INS=Insertion; SNP=single nucleotide polymorphism; TNP=Triple nucleotide polymorphism; CNV=Copy Number Variation. **B,** Oncoprint of *EGFR-RBM10-MET* gene alterations in lung cancer. Targeted whole exome sequencing data from Foundation Medicine was used to display the pattern of co-alterations. **C,** *RBM10* and *EGFR* mutation clonality estimation in the Foundation Medicine dataset. The distribution (kernel density estimation) of mutational allele frequencies of *RBM10* and *EGFR* mutations were analyzed in this clinical dataset (n=45). A threshold for clonality was set at 0.6 (p = 0.02, Fisher exact test) (30). SV=single nucleotide variant, CN=copy number gain.

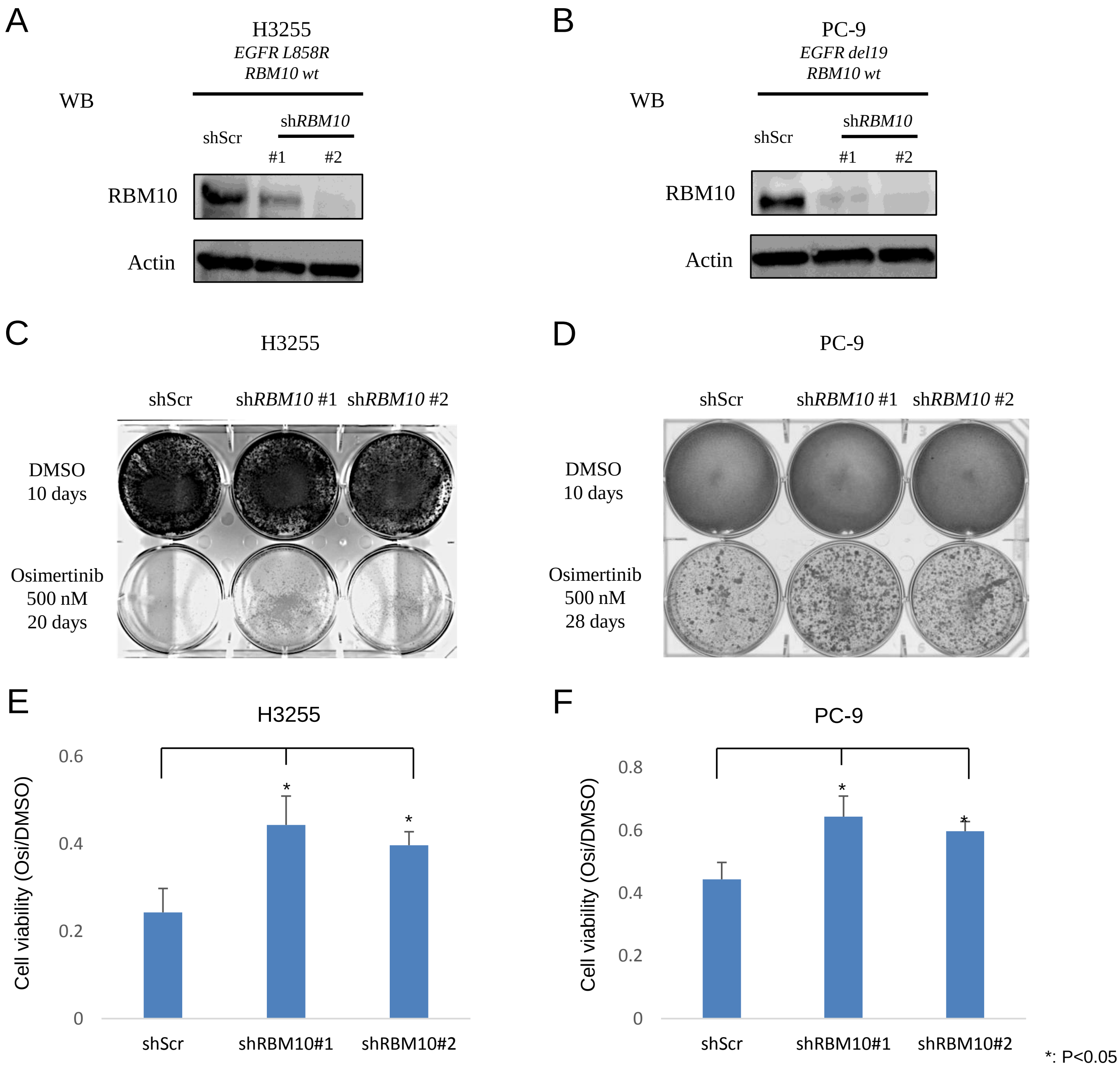
A
H3255
EGFR L858R
RBM10 wt
WB
shScr
shRBM10
#1
#2
RBM10
Actin
B
PC-9
EGFR del19
RBM10 wt
WB
shScr
shRBM10
#1
#2
RBM10
Actin
C
H3255
shScr
shRBM10 #1
shRBM10 #2
DMSO
10 days
Osimertinib
500 nM
20 days
D
PC-9
shScr
shRBM10 #1
shRBM10 #2
DMSO
10 days
Osimertinib
500 nM
28 days
E
H3255
Cell viability (Osi/DMSO)
0.6
0.4
0.2
0
shScr
shRBM10#1
shRBM10#2
F
PC-9
Cell viability (Osi/DMSO)
0.8
0.6
0.4
0.2
0
shScr
shRBM10#1
shRBM10#2
*: P<0.05

Supplementary Figure 2. The effects of osimertinib treatment in RBM10-depleted, *EGFR* mutant cell lines in vitro. A-B, H3255 (**A**) or PC-9 (**B**) cells with shScramble or *RBM10* knockdown confirmed by immunoblot. **C-F,** Crystal violet (CV) cell viability assays demonstrating the effects of osimertinib treatment in each indicated *EGFR* mutant cell line model with *RBM10* knockdown (or control) on growth in vitro. Data represent 3 independent experiments (**C, D**). Bars represent the mean ± SD (**E, F**). One-way ANOVA was used to calculate P values, respectively. *; $p < 0.05$.

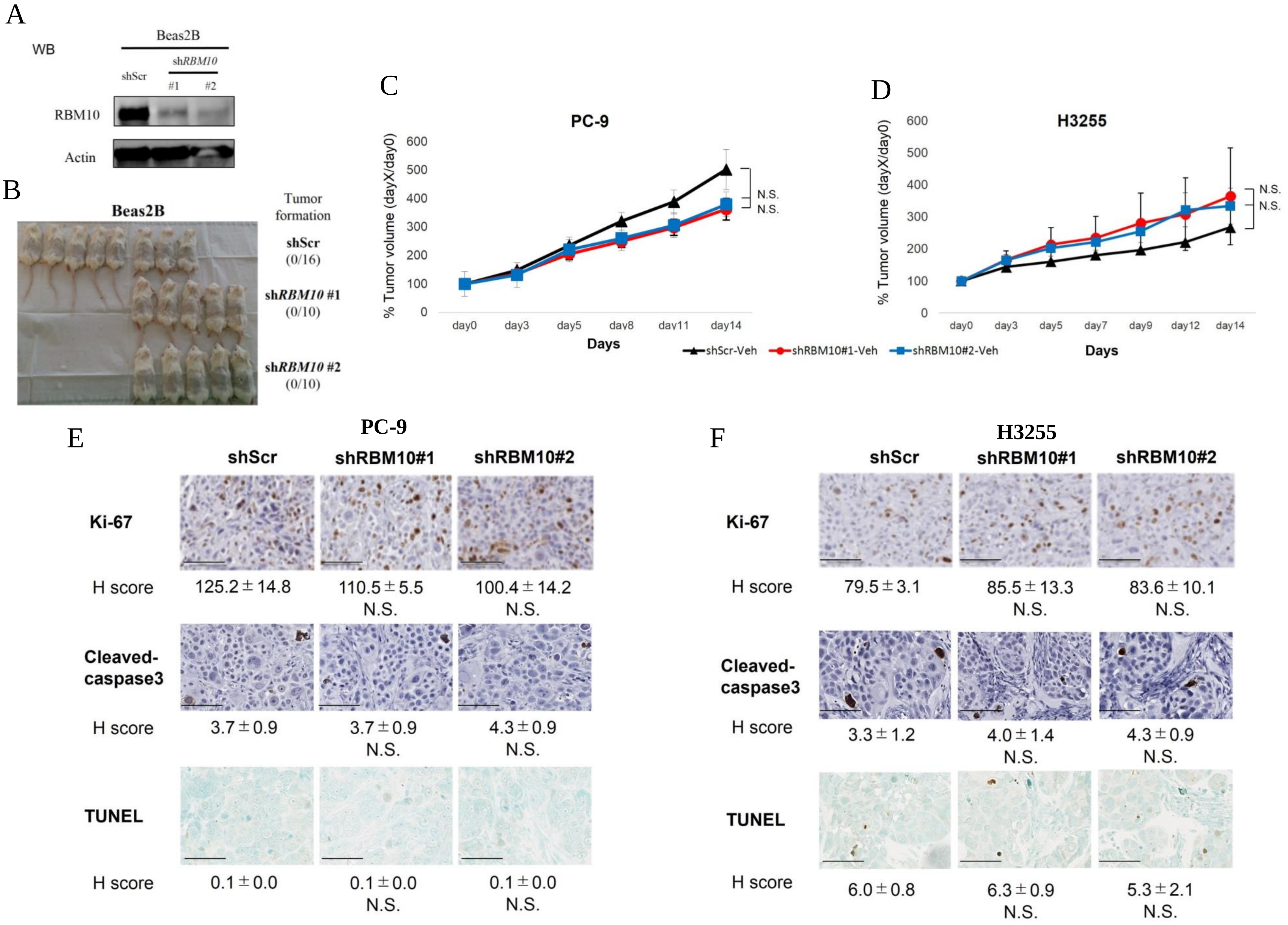

A
WB
Beas2B
shScr
shRBM10
#1
#2
RBM10
Actin
B
Beas2B
Tumor formation
shScr (0/16)
shRBM10 #1 (0/10)
shRBM10 #2 (0/10)
C
PC-9
% Tumor volume (dayX/day0)
600
500
400
300
200
100
0
day0
day3
day5
day8
day11
day14
Days
N.S.
N.S.
D
H3255
% Tumor volume (dayX/day0)
day0
day3
day5
day7
day9
day12
day14
Days
N.S.
N.S.
shScr-Veh
shRBM10#1-Veh
shRBM10#2-Veh
E
PC-9
shScr
shRBM10#1
shRBM10#2
Ki-67
H score
125.2±14.8
110.5±5.5 N.S.
100.4±14.2 N.S.
Cleaved-caspase3
H score
3.7±0.9
3.7±0.9 N.S.
4.3±0.9 N.S.
TUNEL
H score
0.1±0.0
0.1±0.0 N.S.
0.1±0.0 N.S.
F
H3255
shScr
shRBM10#1
shRBM10#2
Ki-67
H score
79.5±3.1
85.5±13.3 N.S.
83.6±10.1 N.S.
Cleaved-caspase3
H score
3.3±1.2
4.0±1.4 N.S.
4.3±0.9 N.S.
TUNEL
H score
6.0±0.8
6.3±0.9 N.S.
5.3±2.1 N.S.

Supplementary Figure 3. The effects of *RBM10* knockdown on tumorigenesis and tumor growth. A-B, Beas2B cells with scramble or *RBM10* knockdown were confirmed with the immunoblot (**A**). Those cells were implanted into immunodeficient mice and observed for 60 days (**B**). **C-F,** SCID mice bearing established tumors with PC-9 (**C, E**) or H3255 (**D, F**) cells with shScramble or *RBM10* knockdown were treated with vehicle control once daily for 14 days. Tumor volume was measured using calipers on the indicated days (n=10 tumors each arm). Percent changes in tumor volume compared to baseline for individual xenografts were measured (**C, D**). Representative IHC analysis of Ki-67, cleaved caspase 3 and TUNEL expression by H-score analysis in PC-9 (E) and H3255 (F) tumors are shown. IHC Scale bars; 100 µm and magnification at 200X. The mean ± SEM for each group is shown. Student's t-test was used to calculate P values, respectively. N.S.: not significant.

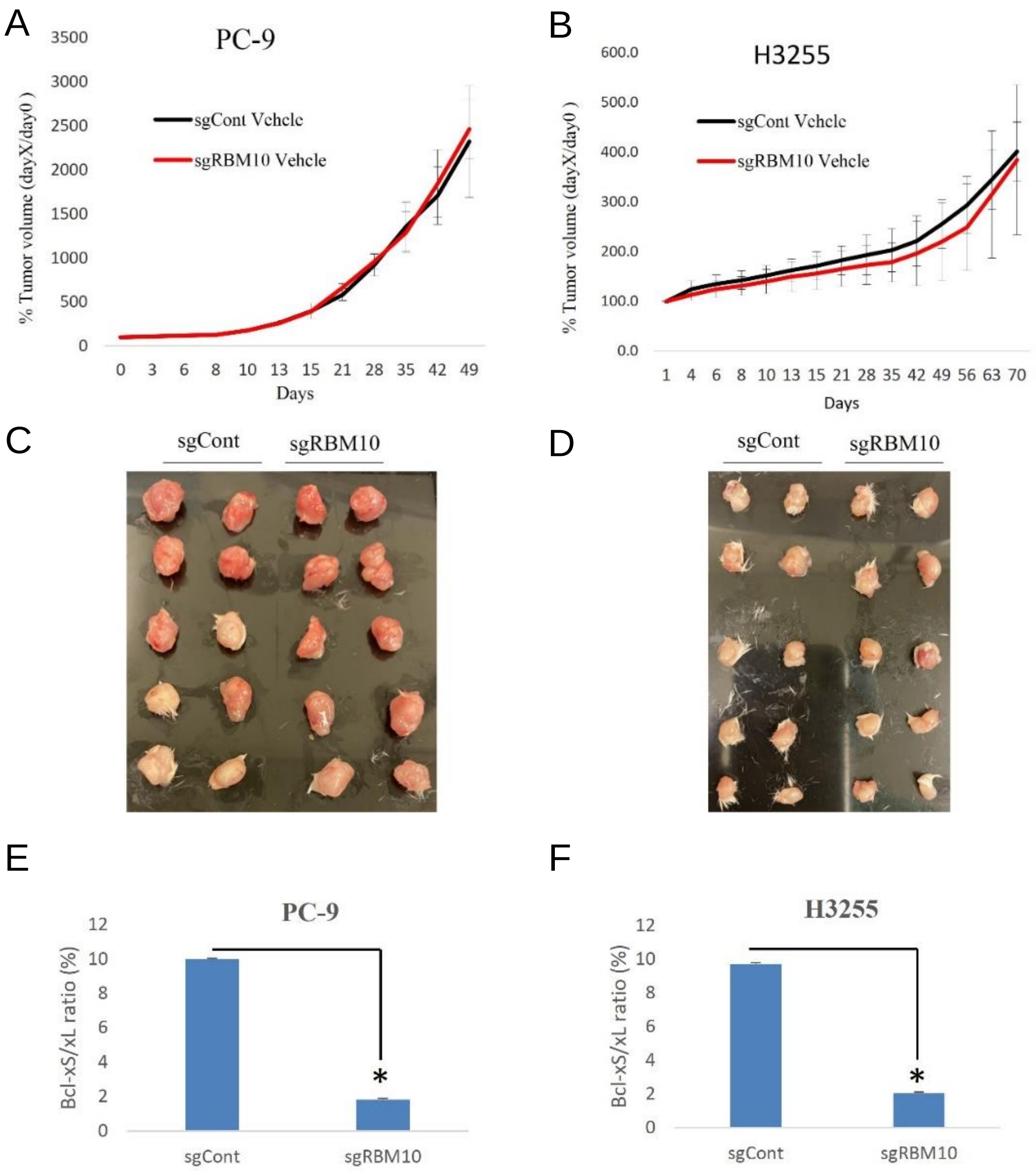
A
PC-9
% Tumor volume (dayX/day0)
sgCont Vchcle
sgRBM10 Vehcle
Days
B
H3255
% Tumor volume (dayX/day0)
sgCont Vchcle
sgRBM10 Vehcle
Days
C
sgCont
sgRBM10
D
sgCont
sgRBM10
E
PC-9
Bcl-xS/xL ratio (%)
sgCont
sgRBM10
F
H3255
Bcl-xS/xL ratio (%)
sgCont
sgRBM10

Supplementary Figure 4. The effects of *RBM10* CRISPR knockout on tumor growth. SCID mice bearing tumors with PC-9 or H3255 cells with sgControl or sg*RBM10* knockout were treated with vehicle control once daily. A-D, Tumor volume was measured using calipers on the indicated days (n=10 tumors each arm). Percent changes in tumor volume compared to baseline for individual xenografts were measured. E-F, The *Bcl-xS/xL* ratio (%) was compared in sgControl (PC-9:10.0, H3255: 9.7) versus sg*RBM10*(PC-9:1.8, H3255: 2.0) tumors by qRT-PCR (*$p<0.01$). Bars represent the mean ± SEM. One-way ANOVA was used to calculate P values, respectively.

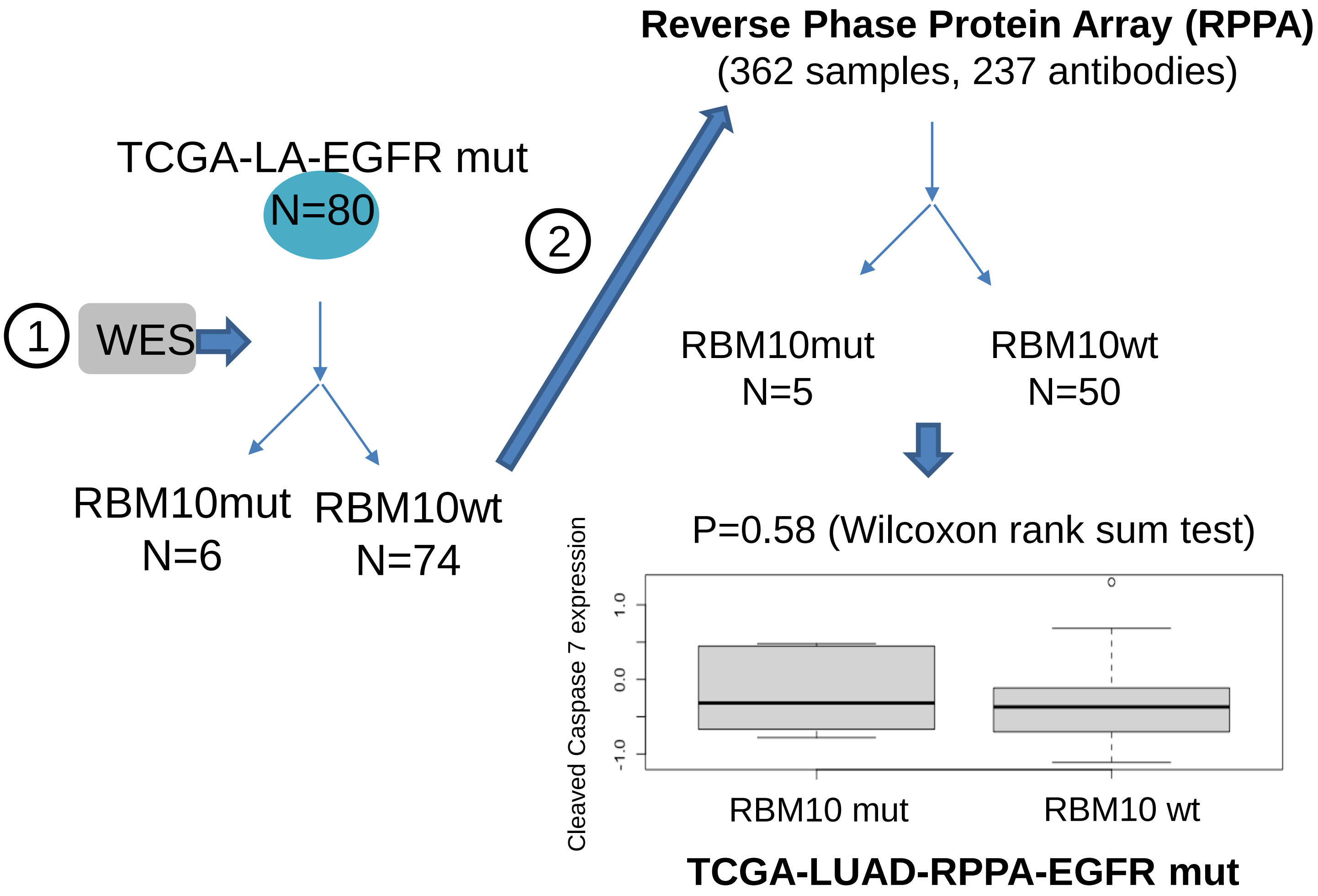

Reverse Phase Protein Array (RPPA)
(362 samples, 237 antibodies)
TCGA-LA-EGFR mut
N=80
2
1
WES
RBM10mut
N=5
RBM10wt
N=50
RBM10mut
N=6
RBM10wt
N=74
P=0.58 (Wilcoxon rank sum test)
Cleaved Caspase 7 expression
1.0
0.0
-1.0
RBM10 mut
RBM10 wt
TCGA-LUAD-RPPA-EGFR mut

Supplementary Figure 5. The effects of *RBM10* mutation on cleaved caspase 7 protein expression. Cleaved Caspase 7 levels were compared between *RBM10* mutant and wild type tumors in Reverse Phase Protein Array (RPPA) data from the TCGA *EGFR* mutant cohort. Wilcoxon rank sum test was used to calculate P values.

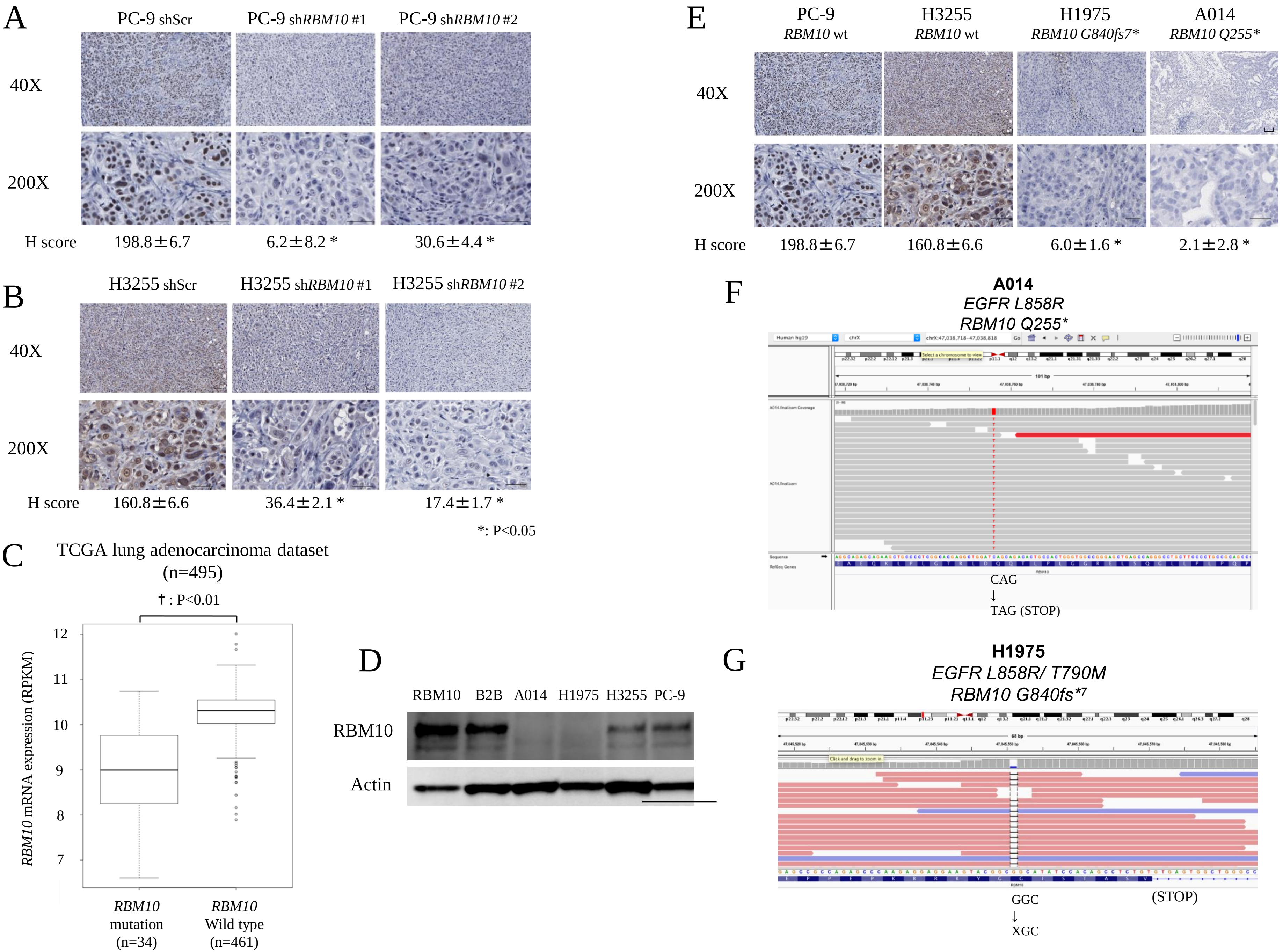
A
PC-9 shScr
PC-9 shRBM10 #1
PC-9 shRBM10 #2
40X
200X
H score
198.8±6.7
6.2±8.2 *
30.6±4.4 *
B
H3255 shScr
H3255 shRBM10 #1
H3255 shRBM10 #2
40X
200X
H score
160.8±6.6
36.4±2.1 *
17.4±1.7 *
*: P<0.05
C
TCGA lung adenocarcinoma dataset
(n=495)
†: P<0.01
RBM10 mRNA expression (RPKM)
12
11
10
9
8
7
RBM10 mutation (n=34)
RBM10 Wild type (n=461)
D
RBM10
B2B
A014
H1975
H3255
PC-9
RBM10
Actin
E
PC-9 RBM10 wt
H3255 RBM10 wt
H1975 RBM10 G840fs7*
A014 RBM10 Q255*
40X
200X
H score
198.8±6.7
160.8±6.6
6.0±1.6 *
2.1±2.8 *
F
A014
EGFR L858R
RBM10 Q255*
CAG
↓
TAG (STOP)
G
H1975
EGFR L858R/ T790M
RBM10 G840fs*7
GGC
↓
XGC
(STOP)

Supplementary Figure 6. RBM10 expression in tumor xenograft and clinical samples. A-B, Representative IHC images of RBM10 in tumors harvested from H3255 and PC-9 subcutaneous tumor xenografts expressing either shScr or sh*RBM10*. **C,** *RBM10* mRNA expression levels in TCGA lung adenocarcinoma samples. *RBM10* mutant and WT samples were stratified using whole exome mutation data and the *RBM10* mRNA expression levels were obtained from corresponding RNAseq data. The boxplot with whisker plot shows the minimum, median, maximum, first and third quantile expression levels. The Y-axis shows the normalized gene expression (RPKM; Reads Per Kilobase Million). **†** : p <0.01. **D,** RBM10 protein expression in a panel of cell lines. **E,** Representative IHC analysis of RBM10 expression and H-score in tumors harvested from subcutaneous tumor xenografts of H3255 and PC-9 (*RBM10* WT) (The shScr in A and B are presented as duplicated images here), and H1975 (*RBM10 G840fs**) and A014 (*RBM10 Q255**) cells. **F-G,** Whole exome sequencing findings for A014 (*RBM10 Q255**) and H1975 (*RBM10 G840fs**) indicating the C to T change and frameshift mutation, respectively. Scale bars; 100 µm and magnification at 40X and 200X. Student's t-test was used to calculate P values, respectively. *; p < 0.05.

A

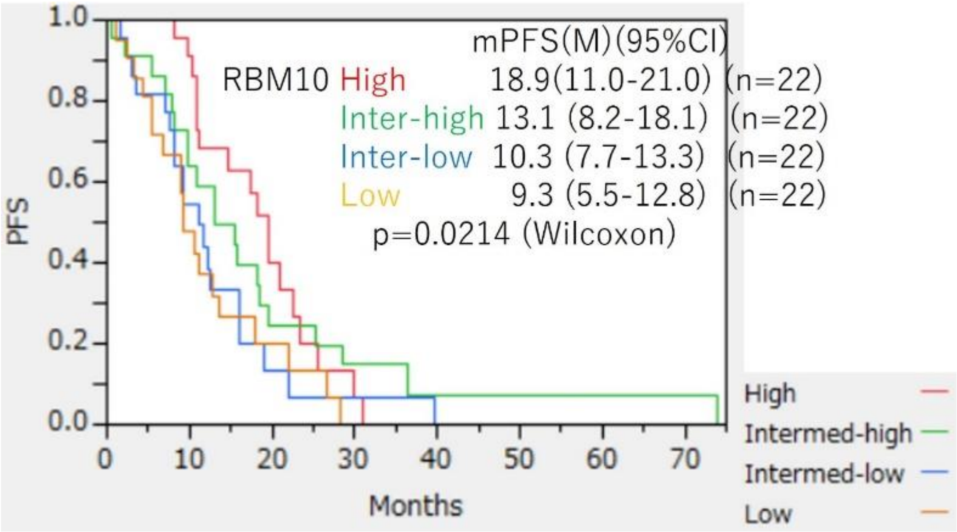
mPFS(M)(95%CI)
RBM10 High 18.9(11.0-21.0) (n=22)
Inter-high 13.1 (8.2-18.1) (n=22)
Inter-low 10.3 (7.7-13.3) (n=22)
Low 9.3 (5.5-12.8) (n=22)
p=0.0214 (Wilcoxon)
PFS
Months
High
Intermed-high
Intermed-low
Low

B

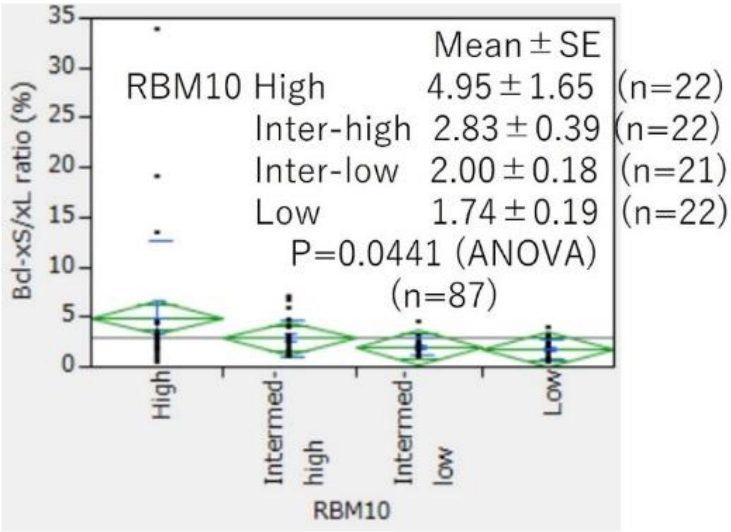
Mean ± SE
RBM10 High 4.95 ± 1.65 (n=22)
Inter-high 2.83 ± 0.39 (n=22)
Inter-low 2.00 ± 0.18 (n=21)
Low 1.74 ± 0.19 (n=22)
P=0.0441 (ANOVA)
(n=87)
Bcl-xS/xL ratio (%)
High
Intermed-high
Intermed-low
Low
RBM10

Supplementary Figure 7. Lower *RBM10* expression is associated with shorter PFS and a decreased *Bcl-xS/xL* ratio in *EGFR* mutant NSCLC. A human EGFR mutant NSCLC, EGFR TKI-treated patient cohort (n=88) was stratified into *RBM10* high to low mRNA expression in quartiles. PFS (**A**: p-value, Wilcoxon test) and the *Bcl-xS/xL* ratio (**B**: p-value, ANOVA) across *RBM10* high to low expression groups are shown.

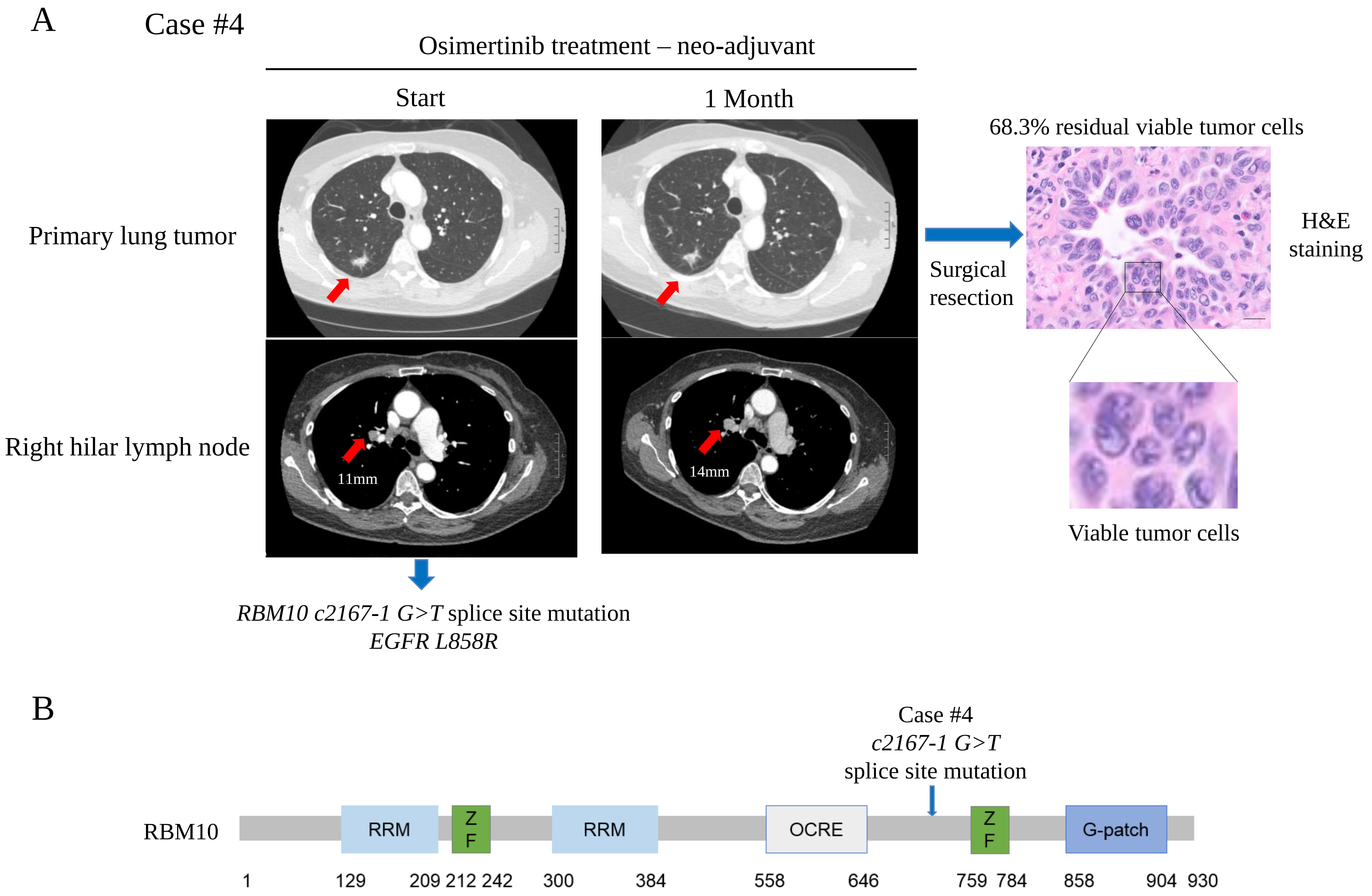
A
Case #4
Osimertinib treatment – neo-adjuvant
Start
1 Month
Primary lung tumor
Right hilar lymph node
11mm
14mm
Surgical
resection
68.3% residual viable tumor cells
H&E
staining
Viable tumor cells
RBM10 c2167-1 G>T splice site mutation
EGFR L858R
B
Case #4
c2167-1 G>T
splice site mutation
RBM10
RRM
Z
F
RRM
OCRE
Z
F
G-patch
1
129
209 212 242
300
384
558
646
759 784
858
904 930

Supplementary Figure 8. *RBM10* mutation is associated with suboptimal EGFR TKI response in *EGFR* mutant NSCLC. A, Case #4 was a patient enrolled in a neo-adjuvant osimertinib clinical trial and found to harbor co-occurring *EGFR L858R* and *RBM10 c2167-1 G>T* splice site mutations. Following one month of osimertinib treatment, radiographic measurements indicated no substantial regression of the primary lung tumor or the enlarged hilar lymph node. Following two months of osimertinib treatment, pathologic evaluation of the resected tumor specimen showed 68.3% residual tumor cell viability by H&E staining (200X magnification). Scale bar; 100 µm. **B,** The position of the *RBM10 c2167-1 G>T* splice site mutation is shown.

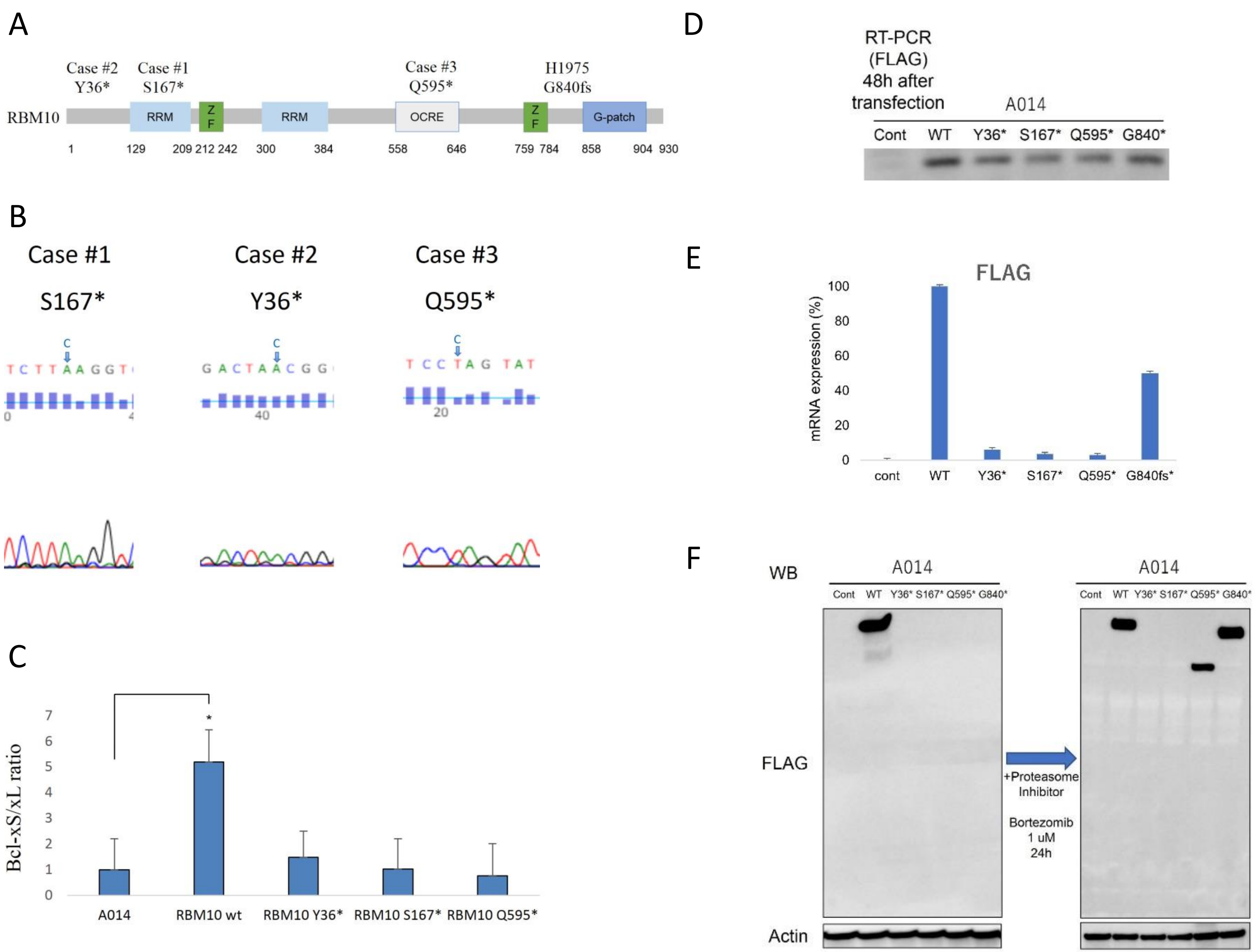
A
Case #2
Y36*
Case #1
S167*
Case #3
Q595*
H1975
G840fs
RBM10
RRM
ZF
RRM
OCRE
ZF
G-patch
1
129
209 212 242
300
384
558
646
759 784
858
904 930
B
Case #1
S167*
Case #2
Y36*
Case #3
Q595*
C
Bcl-xS/xL ratio
*
0
1
2
3
4
5
6
7
A014
RBM10 wt
RBM10 Y36*
RBM10 S167*
RBM10 Q595*
D
RT-PCR
(FLAG)
48h after
transfection
A014
Cont
WT
Y36*
S167*
Q595*
G840*
E
FLAG
mRNA expression (%)
0
20
40
60
80
100
cont
WT
Y36*
S167*
Q595*
G840fs*
F
WB
A014
Cont
WT
Y36*
S167*
Q595*
G840*
FLAG
+Proteasome
Inhibitor
Bortezomib
1 uM
24h
A014
Cont
WT
Y36*
S167*
Q595*
G840*
Actin

Supplementary Figure 9. Effect of clinically annotated *RBM10* mutations on the Bcl-xS/Bcl-xL ratio and biology of RBM10. A, Engineered *RBM10 Y36*, S167*, Q595** and *G840fs** mutations and the known domains of *RBM10* are shown. **B,** RT-PCR analysis of A014 (*RBM10 Q255**) cells transfected with *RBM10* mutations (*Y36**, *S167** and *Q595**) constructs. Mutant sequence is shown, with reference nucleotide in blue. **C,** Quantitative RT-PCR analysis of the Bcl-xS-to-Bcl-xL ratio (mRNA levels) in A014 cells with *RBM10* mutations (*Y36**, *S167** and *Q595**) and WT *RBM10*. **D-E,** RT-PCR analysis (**D**) and quantitative RT-PCR (**E**) analysis of the FLAG (mRNA levels) following genetic reconstitution of N-terminal FLAG tagged *RBM10* mutations in RBM10-deficient A014 cells. F, N-terminal FLAG tagged *RBM10* mutations reconstituted in RBM10-deficient A014 cells under treatment with the proteasome inhibitor Bortezomib at 1μM for 24 hours. Cell lysates were harvested, and the FLAG protein expression was determined by western blot analysis. Data represent 3 independent experiments. Bars represent the mean ± SEM. One-way ANOVA was used to calculate P values. *: $p < 0.05$

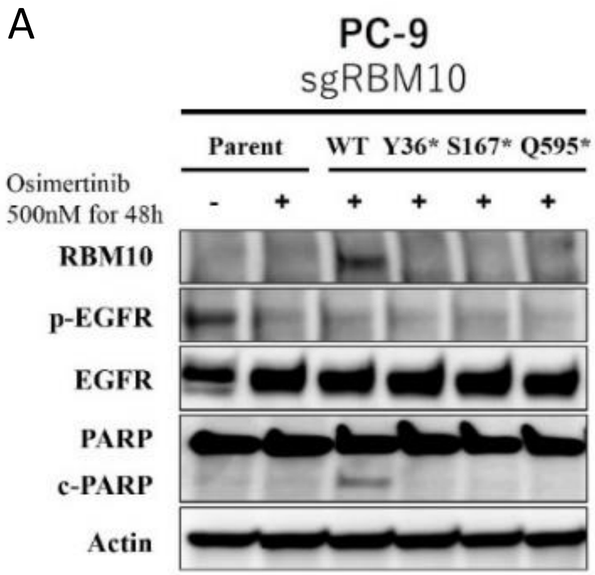
A
PC-9
sgRBM10
Parent
WT
Y36*
S167*
Q595*
Osimertinib
500nM for 48h
-
+
+
+
+
+
RBM10
p-EGFR
EGFR
PARP
c-PARP
Actin

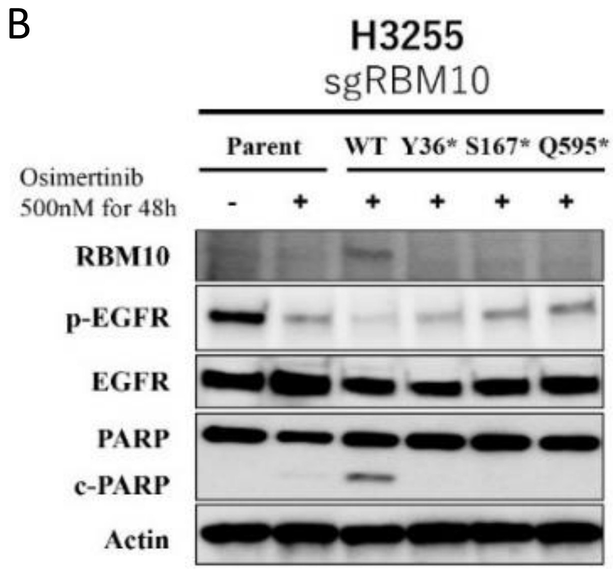
B
H3255
sgRBM10
Parent
WT
Y36*
S167*
Q595*
Osimertinib
500nM for 48h
-
+
+
+
+
+
RBM10
p-EGFR
EGFR
PARP
c-PARP
Actin

Supplementary Figure 10. Effect of clinically annotated *RBM10* mutations on Osimertinib treatment response. Immunoblot analysis of *RBM10* CRISPR knock out PC-9 (**A**) and H3255 (**B**) cells transfected with constructs overexpressing *RBM10* WT or mutant forms (*Y36**, *S167**, *Q595**). Cells were treated with osimertinib (500 nM) or DMSO for 48 hours and western blot analysis for the indicated proteins was performed on cellular extracts.

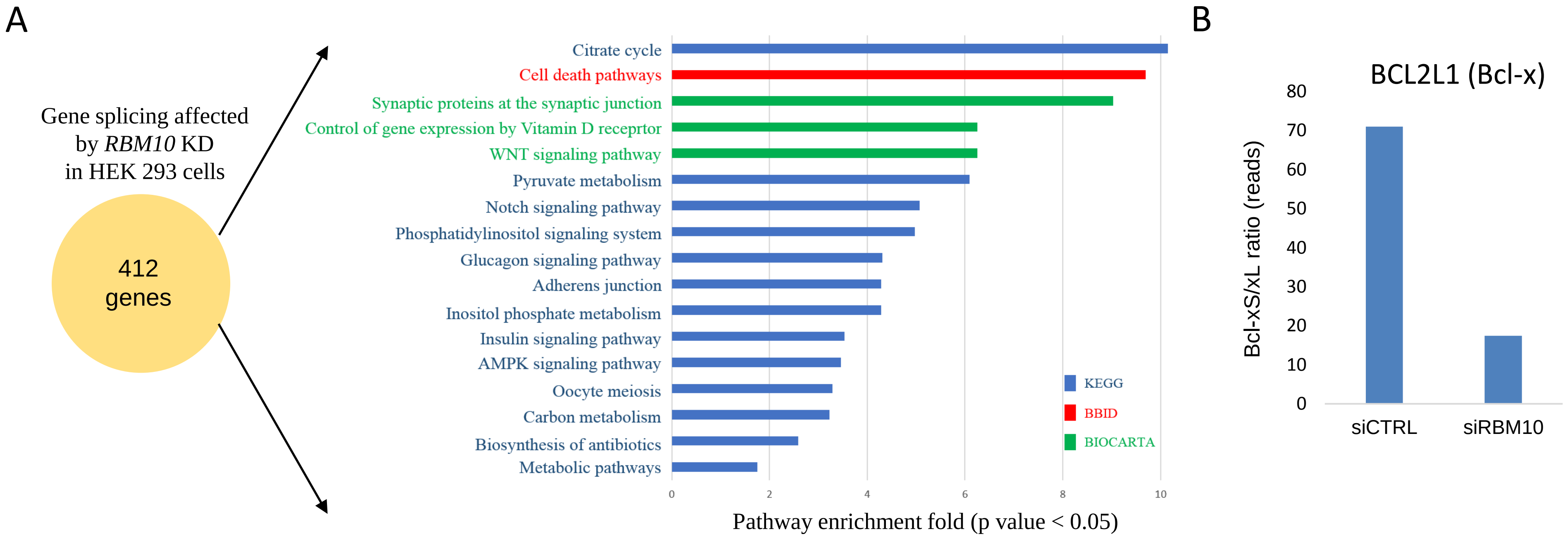
A
Gene splicing affected by *RBM10* KD in HEK 293 cells
412 genes
Citrate cycle
Cell death pathways
Synaptic proteins at the synaptic junction
Control of gene expression by Vitamin D receprtor
WNT signaling pathway
Pyruvate metabolism
Notch signaling pathway
Phosphatidylinositol signaling system
Glucagon signaling pathway
Adherens junction
Inositol phosphate metabolism
Insulin signaling pathway
AMPK signaling pathway
Oocyte meiosis
Carbon metabolism
Biosynthesis of antibiotics
Metabolic pathways
KEGG
BBID
BIOCARTA
0
2
4
6
8
10
Pathway enrichment fold (p value < 0.05)
B
BCL2L1 (Bcl-x)
Bcl-xS/xL ratio (reads)
80
70
60
50
40
30
20
10
0
siCTRL
siRBM10

Supplementary Figure 11. Global analysis of alternative mRNA splicing regulated by *RBM10* in HEK293 cells. A, mRNA splicing of 412 genes is differentially regulated by *RBM10* knockdown (si*RBM10* for 72h) in HEK293 cells. Functional annotation of these differentially spliced genes was carried out using multiple databases (KEGG, BBID and BIOCARTA). The data are represented as a bar diagram indicating probability of fold pathway enrichment. Adjusted p value cutoff for significant gene sets was set to 0.05. Hypergeometric test was used for pathway enrichment analysis within the algorithm (DAVID 6.8). **B,** A trend towards a decrease in the Bcl-xS-to-Bcl-xL ratio, derived from RNA deep sequencing reads, in HEK293 cells with *RBM10* knockdown (si*RBM10*) compared to control (siCTRL) was observed ($p = 0.06$: Student's t-test).

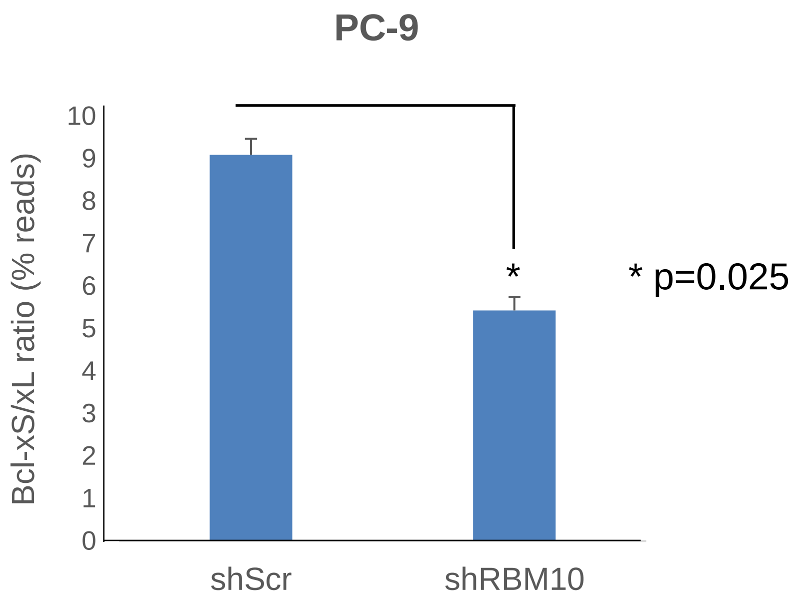
PC-9
Bcl-xS/xL ratio (% reads)
10
9
8
7
6
5
4
3
2
1
0
*
shScr
shRBM10
* p=0.025

Supplementary Figure 12. Bcl-xS/xL ratio change by *RBM10* knockdown in PC-9 cells. Quantitative RT-PCR analysis of the *Bcl-xS*-to-*Bcl-xL* ratio (mRNA levels) in PC-9 cells expressing sh*RBM10* or shScr control ($p<0.05$). Bars represent the mean ± SEM. Student's t-test was used to calculate P values.

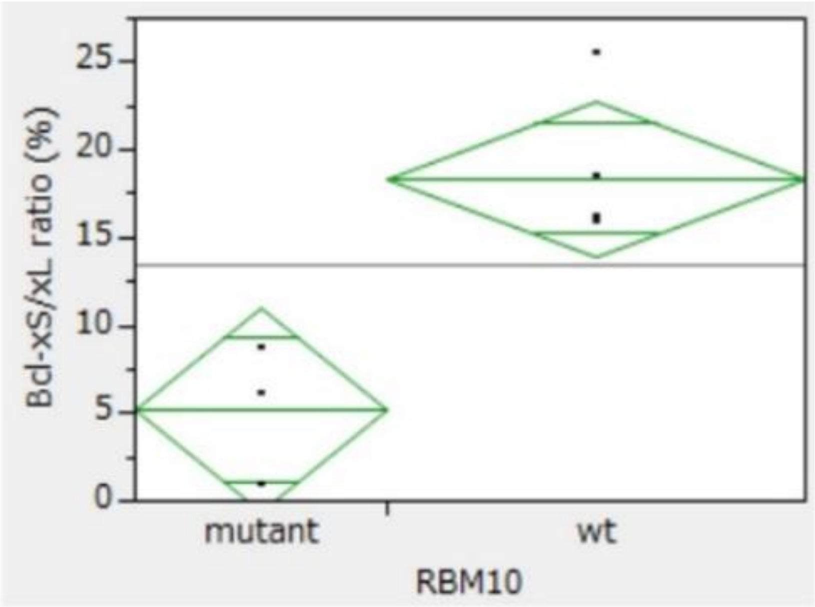

	Mean ± S.E.	
RBM10 mutant	5.19 ± 2.35	(n=3)
wild type	18.4 ± 1.82	(n=5)
	p=0.0045	

Supplementary Figure 13. Effect of *RBM10* mutation on the *Bcl-xS/xL* ratio. The *Bcl-xS/xL* ratio was compared in 5 *RBM10* wild type tumors and 3 mutant tumors from *EGFR* mutant cases (p=0.0045). Student's t-test was used to calculate P values, respectively.

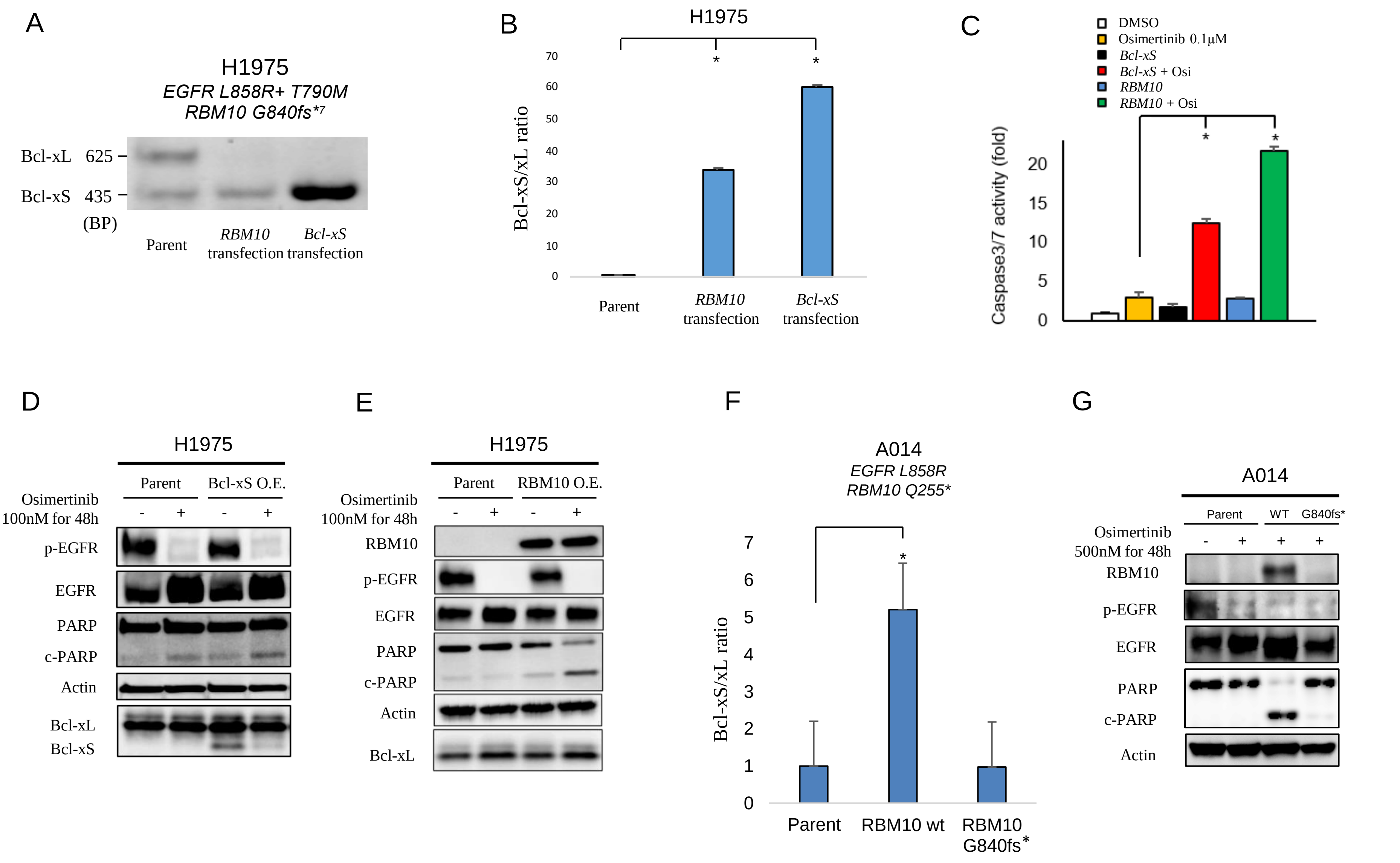
A
H1975
EGFR L858R+ T790M
RBM10 G840fs*7
Bcl-xL 625
Bcl-xS 435
(BP)
Parent
RBM10 transfection
Bcl-xS transfection
B
H1975
Bcl-xS/xL ratio
70
60
50
40
30
20
10
0
*
*
Parent
RBM10 transfection
Bcl-xS transfection
C
DMSO
Osimertinib 0.1μM
Bcl-xS
Bcl-xS + Osi
RBM10
RBM10 + Osi
Caspase3/7 activity (fold)
20
15
10
5
0
*
*
D
H1975
Parent
Bcl-xS O.E.
Osimertinib 100nM for 48h
- + - +
p-EGFR
EGFR
PARP
c-PARP
Actin
Bcl-xL
Bcl-xS
E
H1975
Parent
RBM10 O.E.
Osimertinib 100nM for 48h
- + - +
RBM10
p-EGFR
EGFR
PARP
c-PARP
Actin
Bcl-xL
F
A014
EGFR L858R
RBM10 Q255*
Bcl-xS/xL ratio
7
6
5
4
3
2
1
0
*
Parent
RBM10 wt
RBM10 G840fs*
G
A014
Parent
WT
G840fs*
Osimertinib 500nM for 48h
- + + +
RBM10
p-EGFR
EGFR
PARP
c-PARP
Actin

Supplementary Figure 14. Phenotype of the RBM10-deficient H1975 cell line. A, Conventional PCR analysis using validated primers to detect both *Bcl-xL* and *Bcl-xS* isoforms in H1975 cells with genetic rescue of *RBM10* or *Bcl-xS* compared to parental control is shown. **B,** Quantitative RT-PCR analysis of the Bcl-xS-to-Bcl-xL ratio (mRNA levels) in H1975 cells with genetic rescue of *RBM10* or *Bcl-xS* compared to parental control cells. **C, D, E,** H1975 cells (RBM10-deficient) overexpressing *Bcl-xS* or reconstituted with *RBM10* 24 hours before treatment with osimertinib (100 nM) or DMSO control. The activity of Caspase-3/7 was measured using Caspase-Glo 3/7 assay. Each bar represents the mean ± SEM of the fold change after normalization to DMSO control (**C**). Cell lysates were harvested, and the indicated proteins were determined by western blot analysis (**D, E**). **F,** Quantitative RT-PCR analysis of the Bcl-xS-to-Bcl-xL ratio (mRNA levels) in A014 (*RBM10 Q255**) cells expressing *RBM10* WT or the *RBM10* mutation native to H1975 ($G840fs*^{7}$). **G,** Immunoblot analysis of A014 cells transfected with expression constructs containing *RBM10* WT or the H1975 *RBM10* variant ($G840fs*^{7}$). Cells were treated with osimertinib (500 nM) or DMSO for 48 hours and western blot analysis was performed on cellular extracts. Data represent 3 independent experiments. O.E.: Overexpression. BP; Base Pairs. One-way ANOVA was used to calculate P values, respectively. *; $p < 0.05$.

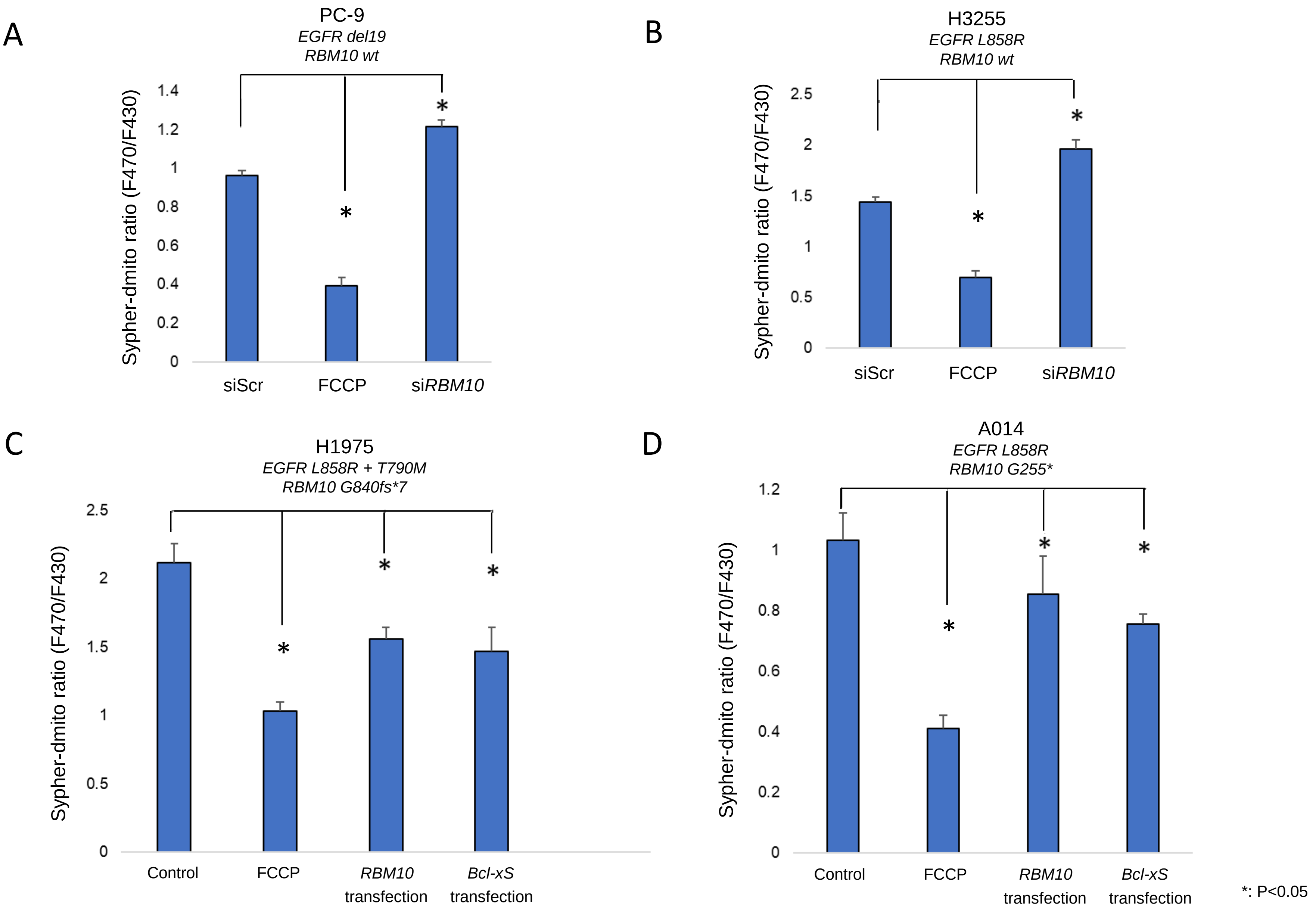
A
PC-9
EGFR del19
RBM10 wt
Sypher-dmito ratio (F470/F430)
1.4
1.2
1
0.8
0.6
0.4
0.2
0
*
*
siScr
FCCP
siRBM10
B
H3255
EGFR L858R
RBM10 wt
Sypher-dmito ratio (F470/F430)
2.5
2
1.5
1
0.5
0
*
*
siScr
FCCP
siRBM10
C
H1975
EGFR L858R + T790M
RBM10 G840fs*7
Sypher-dmito ratio (F470/F430)
2.5
2
1.5
1
0.5
0
*
*
*
Control
FCCP
RBM10 transfection
Bcl-xS transfection
D
A014
EGFR L858R
RBM10 G255*
Sypher-dmito ratio (F470/F430)
1.2
1
0.8
0.6
0.4
0.2
0
*
*
*
Control
FCCP
RBM10 transfection
Bcl-xS transfection
*: P<0.05

Supplementary Figure 15. Change of mitochondrial membrane potential in response to RBM10 differential expression. A-D, Cells were stably expressed with SypHer-dmito construct. Cells were treated with uncoupling reagent carbonyl cyanide 4-(trifluoromethoxy) phenylhydrazone (FCCP) as a positive control. SiScramble or si*RBM10* conditions are shown for PC-9 (**A**) and H3255 (**B**) cell lines. RBM10 or Bcl-xS expression constructs for H1975 (**C**) and A014 (**D**) cell lines were transfected 48 hours before image acquisition. SypHer-dmito fluorescence (F470/F430) ratio was calculated for each cell and analyzed with ImageJ software. Each bar represents the mean ± SEM. One-way ANOVA was used to calculate P values, respectively. *: $p < 0.05$ compared to control of each cell.

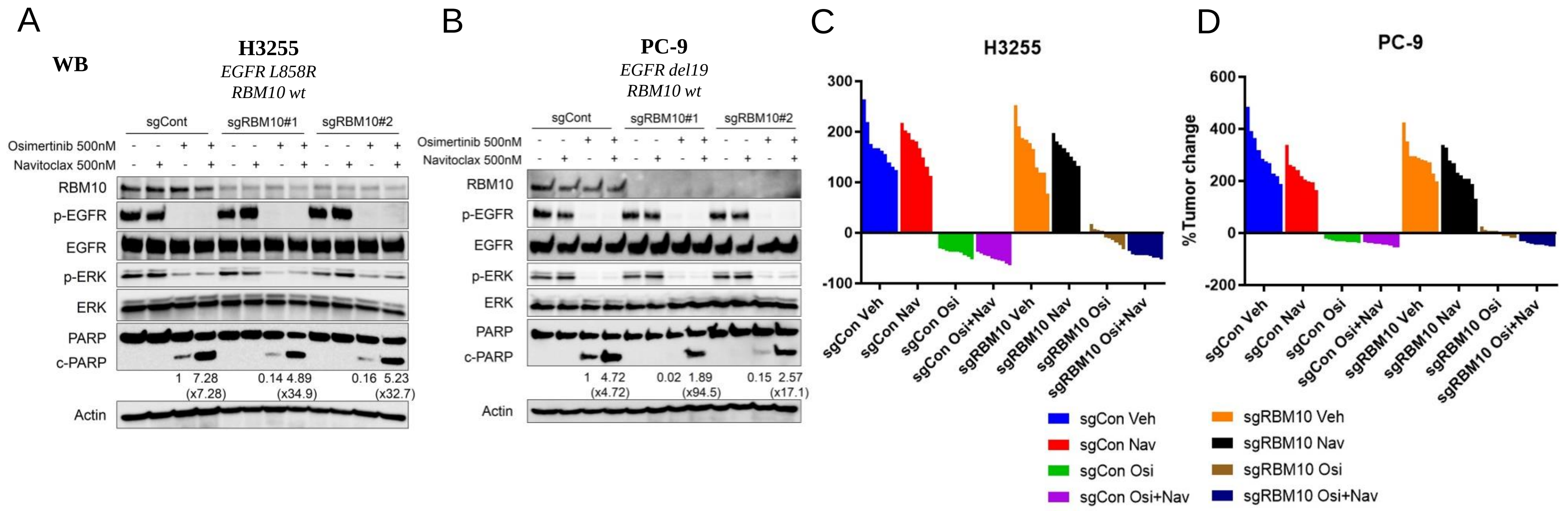

E

H3255	Vehcle		Navitoclax		Osimertinib		Osi + Nav	
	sgCont	sgRBM10	sgCont	sgRBM10	sgCont	sgRBM10	sgCont	sgRBM10
CR	0	0	0	0	0	0	0	0
PR	0	0	0	0	10	1	10	10
SD	0	0	0	0	0	9	0	0
PD	10	10	9	8	0	0	0	0
RR (%)	0	0	0	0	100	10	100	100

N.S.
p=0.0001

F

PC-9	Vehcle		Navitoclax		Osimertinib		Osi + Nav	
	sgCont	sgRBM10	sgCont	sgRBM10	sgCont	sgRBM10	sgCont	sgRBM10
CR	0	0	0	0	0	0	0	0
PR	0	0	0	0	8	0	10	10
SD	0	0	0	0	2	9	0	0
PD	10	10	9	10	0	1	0	0
RR (%)	0	0	0	0	80	0	100	100

N.S.
P<0.0001

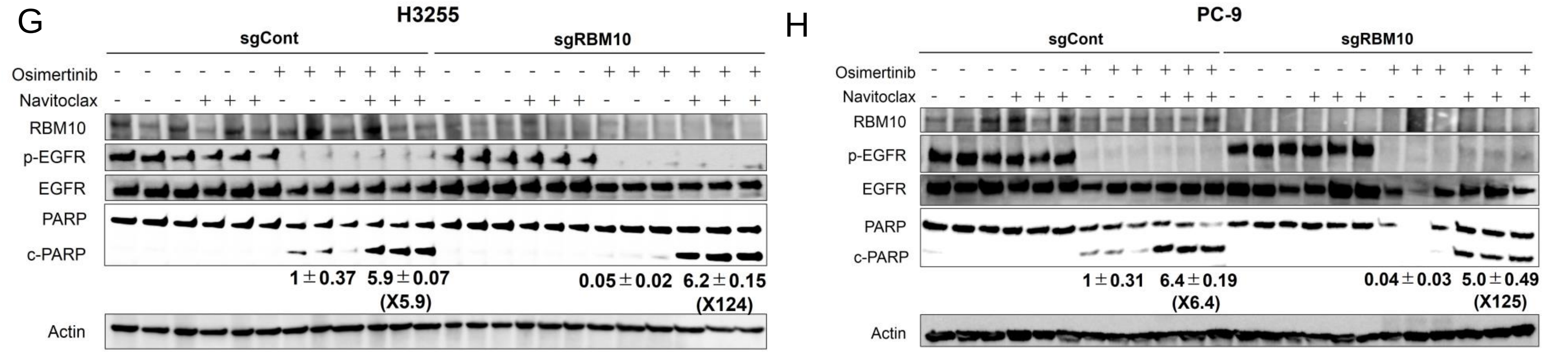

Supplementary Figure 16. Combined Navitoclax effects with Osimertinib in RBM10-depleted *EGFR* mutant cell lines and tumors. A-B, Western blot analysis of Navitoclax (ABT-263) 500 nM, in combination with osimertinib 500 nM treatment in H3255 ($EGFR^{L858R}$; $RBM10^{\mathrm{WT}}$), PC-9 ($EGFR^{del19}$; $RBM10^{\mathrm{WT}}$) models with sgControl (sgCont) or sg*RBM10*. **C-D,** Waterfall plots representing immunodeficient mice bearing H3255 (**C**) or PC-9 (**D**) tumor xenografts expressing either sgCont or sg*RBM10*; mice were treated with vehicle, navitoclax (50mg/kg), 2 mg/kg (H3255) or 5mg/kg (PC-9) osimertinib, or combination (navitoclax and osimertinib) therapy for 14 days (n=10 tumors each arm). Percent changes in tumor volume compared to baseline volume (Day 0) for individual tumor xenografts are shown. **E-F,** Objective tumor responses were graded using RECIST response criteria. **G-H,** H3255 (**G**) and PC-9 (**H**) tumor xenograft explants demonstrating the effect of *RBM10* knockout on PARP cleavage in mice treated with indicated treatment for 4 days. Three tumors per group were harvested 4 hours after indicated treatments on day5, and subsequent analyses of the indicated proteins was performed by western blot. CR: Complete response, PR: Partial response, SD: Stable disease, PD: Progression disease, NE: Not evaluable, RR: Response rate. Fisher's exact test was used to calculate P values, respectively.

Sanger Seq　Erlotinib resistance (ER)

Exon20 T790M(ACG⇒ATG)

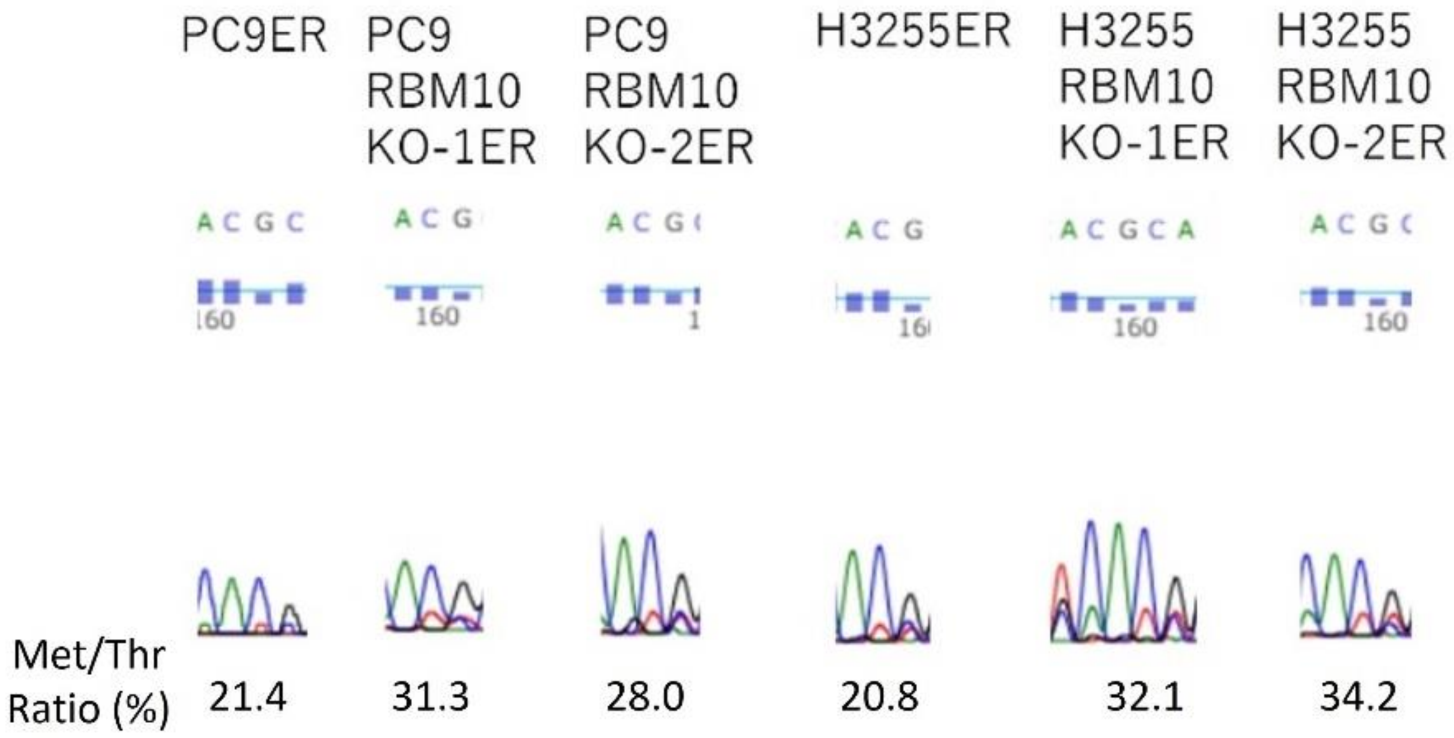

Supplementary Figure 17. EGFR TKI resistance mechanism in RBM10-depleted *EGFR* mutant cells. The *EGFR T790M* mutation sequences in the Erlotinib resistant PC9sgRBM10 and H3255sgRBM10 cells after 2 months of treatment in vitro are shown. The ratio of Methionine to Threonine are shown below the peaks.

	RBM10 low	RBM10 inter-low	RBM10 inter-high	RBM10 high	p value
**Age (Mean ± SD)**	64.1 ± 12.3	63.0 ± 12.2	59.3 ± 13.4	62.3 ± 8.9	N.S.
**Gender (Male / Female)**	4 / 18	6 / 16	7 / 15	3 / 19	N.S.
**EGFR-TKI (Gefitinib / Erlotinib)**	11 / 11	10 / 12	12 / 10	9 / 13	N.S.
**EGFR mutation (Del19 / L858R)**	13 / 6	12 / 7	11 / 11	14 / 8	N.S.
**Brain metastasis (+ / -)**	4 / 18	9 / 13	7 / 15	9 / 13	N.S.

Supplementary Table 1. Clinical parameters of a human EGFR TKI-treated patient cohort (n=88). Available clinical parameters were examined, and no significant differences were found between the groups stratified by clinical parameters in Supplementary Figure 7 (Kruskal-Wallis test). N.S.: not significant.

	RBM10 **wild type**	***RBM10*** **mutant**	**p value**
**Age (Mean ± SD)**	66.2 ± 12.1	66.2 ± 12.1	N.S.
**Gender (Male / Female)**	23 / 34	5 / 8	N.S.
**EGFR-TKI (1st generation / 2nd 3rd generation)**	38 / 19	9 / 4	N.S.
**EGFR mutation (Del19 / L858R)**	30 / 27	4 / 9	N.S. (p=0.13)
**TP53 mutation (+ / -)**	22 / 35	6 / 7	N.S.
**RB1 or CDK4/6 or WNT alt (+ / -)**	46 / 11	12 / 1	N.S. (p=0.44)

Supplementary Table 2. Clinical and co-mutation parameters of a human EGFR TKI-treated patient cohort with WES (n=70). Available co-mutation and clinical data were examined, and no significant differences were found between the groups (Mann-Whitney test). N.S.: not significant.

Supplementary Table 3. Differentially spliced exons upon *RBM10* knockdown. RefSeq exons with significant Percentage Splicing In (PSI) changes (FDR ≤ 5%, ΔPSI ≥ 0.1) after *RBM10* KD in HEK-293 cells (36). Significant Z-values are shown in bold. PSI values in HEK are median between replicates.

Supplementary Table 4. Analysis of RBM10-mediated differential alternative mRNA isoforms in PC-9 lung cancer cells. One hundred eighty-two mRNA isoforms of 171 genes are differentially regulated by *RBM10* knockdown (sh*RBM10*) in *EGFR* mutant PC-9 lung cancer cells. The differential mRNA isoform expression criteria were set as |log2FC | > 1 and FDR < 0.05.
